# Improved Shear Strength Prediction Model of Steel Fiber Reinforced Concrete Beams by Adopting Gene Expression Programming

**DOI:** 10.3390/ma15113758

**Published:** 2022-05-24

**Authors:** Moiz Tariq, Azam Khan, Asad Ullah, Javad Shayanfar, Momina Niaz

**Affiliations:** 1 NUST Institute of Civil Engineering (NICE), School of Civil and Environmental Engineering, National University of Science and Technology (NUST), Sector H-12, Islamabad 44000, Pakistan; azam.khan@nice.nust.edu.pk (A.K.); aullah1.ms18nice@student.nust.edu.pk (A.U.); 2 Department of Civil Engineering, University of Minho, Azur’em, 4800-058 Guimaraes, Portugal; arch3d.ir@gmail.com; 3 Department of Civil Engineering, University of Engineering and Technology, Peshawar 25130, Pakistan; momina.niaz7@gmail.com

**Keywords:** gene expression programming, reinforced concrete, steel fiber reinforced concrete, shear strength

## Abstract

In this study, an artificial intelligence tool called gene expression programming (GEP) has been successfully applied to develop an empirical model that can predict the shear strength of steel fiber reinforced concrete beams. The proposed genetic model incorporates all the influencing parameters such as the geometric properties of the beam, the concrete compressive strength, the shear span-to-depth ratio, and the mechanical and material properties of steel fiber. Existing empirical models ignore the tensile strength of steel fibers, which exercise a strong influence on the crack propagation of concrete matrix, thereby affecting the beam shear strength. To overcome this limitation, an improved and robust empirical model is proposed herein that incorporates the fiber tensile strength along with the other influencing factors. For this purpose, an extensive experimental database subjected to four-point loading is constructed comprising results of 488 tests drawn from the literature. The data are divided based on different shapes (hooked or straight fiber) and the tensile strength of steel fiber. The empirical model is developed using this experimental database and statistically compared with previously established empirical equations. This comparison indicates that the proposed model shows significant improvement in predicting the shear strength of steel fiber reinforced concrete beams, thus substantiating the important role of fiber tensile strength.

## 1. Introduction

The concept of fiber-reinforced building materials is by no means new, ancient West Asia, Africa, and South America were the earliest civilizations that were familiar with reinforcing adobe bricks with straw fibers [1]. Much development has taken place since, especially over the past 100 years, when the field of fiber-reinforced concrete has witnessed a boom. Presently, four primary types of fibers—namely, natural, glass, synthetic, and steel—are used in concrete. It follows that the natural fibers (coconut, bamboo, sisal, jute, etc.) are cheap, but they can cause durability issues in concrete [2]. Similarly, glass fiber is prone to durability issues. On the other hand, synthetic fibers (such as nylon, carbon, acrylic, and polyester) can successfully make concrete more durable; however, they do not improve the shear strength as efficiently as steel fibers [1].

Shear failure is one of the most dangerous types of failure, due to its inherent uncertainty and catastrophic nature. In order to avoid this sudden and explosive failure, typical vertical web reinforcement is provided that is spaced at varying intervals. However, the provision of web reinforcement can be challenging if the available space is narrow, irregular, and congested. In such cases, as pointed out earlier, steel fiber reinforcement can be used to effectively increase the shear strength. Although the use of steel fibers, such as metal chips and screws, started as early as 1910 [1], systematic experimental studies in this context appear to have been made in the 1960s. These fibers are more effective when their size ranges from 6.4 mm to 76 mm in length by virtue of being uniformly distributed throughout a concrete matrix [3]. Consequently, this uniform mixing has shown significant improvement in the compressive strength of the concrete; additionally, steel fiber reinforced concrete (SFRC) has shown significant promise in the field of civil engineering, for example, these fibers have been successfully employed in dams, bridge decks, and RC beam-column joints in seismically active regions [4]. The use of steel fiber reinforced (steel fiber reinforced concrete (SFRC)) concrete has not only improved the shear strength of structural elements but also offered beneficial restraining effects to prohibit crack opening. Along with toughness, the contribution of steel fiber reinforced (steel fiber reinforced concrete (SFRC)) concrete has been shown to improve the concrete tensile strength in splitting planes, thus enhancing the dowel resistance [5,6].

Congestion of reinforcing bars at beam-column intersection and placement of concrete in such a region has been a challenging problem for a long time. Steel fiber reinforced concrete (SFRC) has been suggested as one of the potential alternatives. Wang and Lee [5] have shown an improvement in the flexural shear capacity of interior beam-column joints using steel fiber reinforced (steel fiber reinforced concrete (SFRC)) concrete jacketing. Similarly, Filiatrault et al. [7] have shown that the use of steel fiber reinforced (steel fiber reinforced concrete (SFRC)) concrete in the RC joint region not only enhances the ductility of the joint but also substantially increases the joint shear strength. By virtue of this, Fattuhi and Hughues [8] have shown an increased strength and ductility of steel fiber reinforced (steel fiber reinforced concrete (SFRC)) concrete corbels. As a consequence of the ductility, cohesion, and energy absorption property, steel fiber reinforced (steel fiber reinforced concrete (SFRC)) concrete is used as a primary component in shotcrete and other slope stabilization applications [9].

Building design codes such as ACI has encouraged the use of steel fiber as a replacement for minimum stirrups [10]. It may, therefore, be argued that there is a clear need for an accurate procedure for concrete structures reinforced with steel fibers. Due to the complex shear transfer mechanisms of steel fiber reinforced (steel fiber reinforced concrete (SFRC)) concrete and the orientation of the fibers at the crack interface, there is no forthright method of accurate prediction [11]. As a result, empirical formulations have been developed by several researchers (Sharma [12], Kwak et al. [13], Wright [14], Zsutty [15]). However, the major drawback of all the available models is their restricted validity due to the limited dataset employed in the model development.

Recently, a new technique of machine learning models has been able to predict the mechanical properties of concrete with higher accuracy. Several evolutionary algorithms for the shear capacity predictions of steel fiber reinforced (SFRC) concrete beams are proposed by different researchers. For instance, two models using multi-expression programming are proposed by Sarveghadi et al. [16]. Greenough and Nehdi [17] have used a genetic algorithm (GA) to develop a model incorporating a parameter for dowel action. Shahnewaz and Alam [18] have identified various influencing parameters, which are incorporated in a genetic algorithm-based equation for steel fiber reinforced (SFRC) concrete beams. Although all these artificial intelligence models show satisfactory accuracy, the database employed to generate these modes is relatively small [19,20,21]. One other important issue that remains with these models is that they do not consider the mechanical properties of steel fibers. The growing number of available experimental data and the recent advancement of data analysis techniques have motivated new approaches.

The study is motivated by a similar investigation by Sabetifar et al. [22], where a GEP model was proposed to estimate the ultimate shear strength of steel fiber reinforced concrete (SFRC) beams. This work has been extended to enhance the experimental dataset of 488 lab tests and incorporate additional influencing factors such as the diameter of aggregate and, most importantly, the tensile strength of the fiber [23]. It is shown that the tensile strength is one of the most important factors influencing the shear strength of the steel fiber reinforced concrete (SFRC) beam. This conclusion is drawn from a comprehensive statistical comparison between the current model and fifteen existing models.

## 2. Research Significance

The shear failure of a structural element built from concrete is very dangerous because of the greater uncertainty and the catastrophic nature of failure [24]. This has inspired many researchers to examine the use of steel fibers for improving the shear strength of beams. Several empirical models have been proposed for evaluating the shear strength of steel fiber reinforced concrete (SFRC) beams. However, most of these approaches are either computationally demanding or too approximate. While these models incorporate most of the key influencing parameters, they tend to omit the most important factor of fiber tensile strength [23]. The current study attempts to develop an accurate model by incorporating the fiber tensile strength in addition to other influencing factors [25], thus a substantial improvement on the existing shear models.

## 3. Factors Affecting Shear Strength

Identifying key influencing parameters is the most important step toward generating an accurate model. The influencing factors chosen for modeling the shear behavior of steel fiber reinforced concrete (SFRC) beams herein are the concrete compressive strength, the tensile reinforcement ratio, the span to depth ratio, the fiber aspect ratio, the number of fibers in concrete, the fiber tensile strength, and the span of a beam. Of special interest in these parameters is the fiber tensile strength, which has been overlooked by all the previous studies [3,26,27,28,29].

Khuntia et al. [27] have shown that the increase in the concrete compressive strength results in an exponential increase in the shear strength of steel fiber reinforced concrete (SFRC) beams, which has been attributed to the development of the strong bond between the concrete matrix and fibers [28]. Nevertheless, owing to the arching action, an exponential decrease in the beam shear strength is observed with an increase in the shear span-depth ratio [3]. Another important factor of shear strength is the tensile reinforcement ratio, whose increase increases the shear strength up to a certain limit beyond which the strength declines [29]. It has been suggested that lower reinforcement ratios result in larger dowel forces in concrete, and an increased shear strength ensues [30]. On the other hand, the rate of increase in these beams decreases when the reinforcement ratios increase.

Finally, the effect of steel fiber tensile strength has a significant influence on the steel fiber reinforced concrete (SFRC) beams. Studies have shown that the toughness and the shear strength of the RC beam can be increased by improving the tensile strength of the steel fiber [3]. Moreover, the improved tensile strength not only enhances the ductility of the structural element, resulting in high energy dissipation but also increases the fiber bridging effect, causing a delay in the propagation of cracks.

## 4. Parametric Study

**Figure 1:**
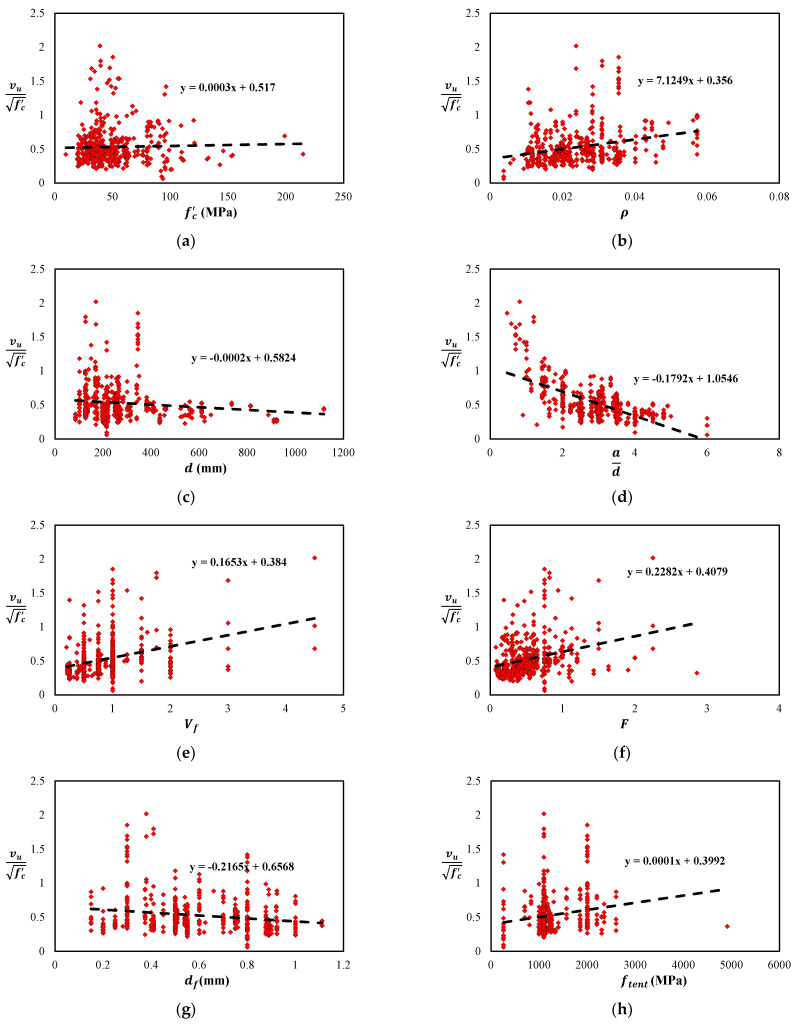

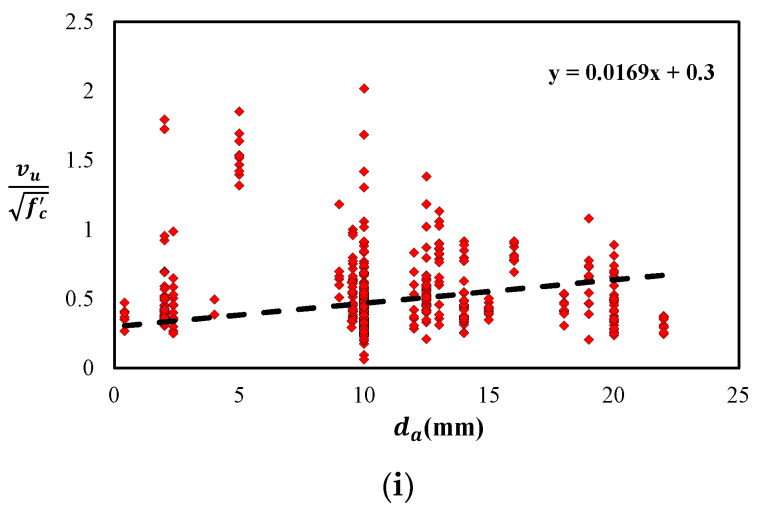
Factors influencing the shear strength of steel fiber reinforced (steel fiber reinforced concrete (SFRC)) concrete beam: (**a**) Concrete compressive strength, (**b**) Reinforcement ratio, (**c**) Effective depth, (**d**) Span to depth ratio, (**e**) Fiber volume fraction, (**f**) Fiber factor, (**g**) Fiber diameter, (**h**) Tensile strength of fiber, (**i**) Diameter of aggregate.

**Figure 2:**
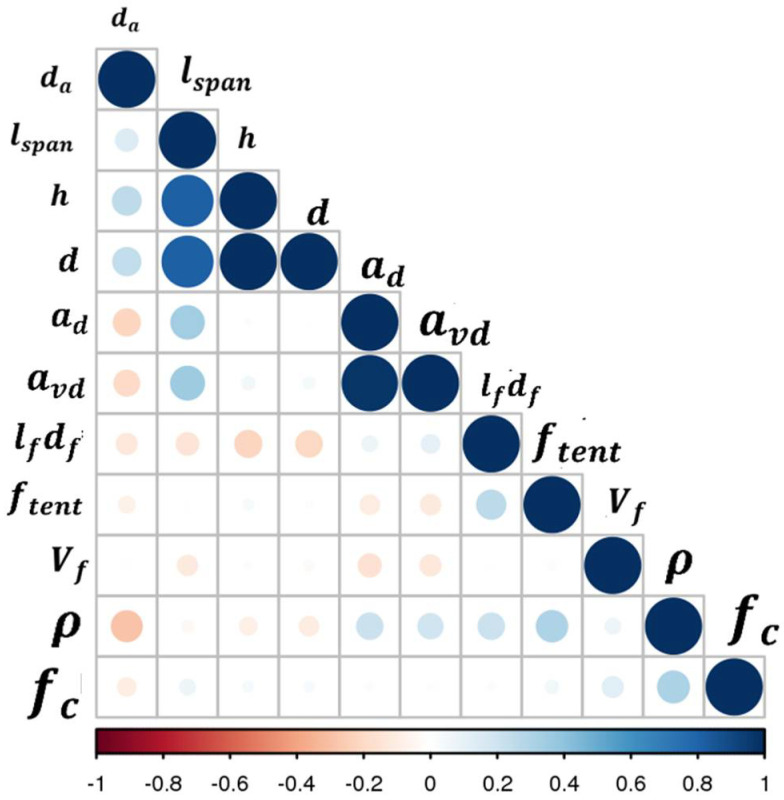
Correlation between input parameters.

This section is concerned with a parametric study performed to enhance the understanding of important factors influencing the shear strength of steel fiber reinforced concrete (SFRC) beams. The aim is to create a set of circumstances across which only one parameter, affecting the outcome of interest, varies. To achieve this objective in the current parametric study, it is essential to make concrete compressive strength uniform, so that its confounding effect is minimized. Therefore, the shear strength values are normalized with respect to the concrete compressive strength to create a circumstance in which the effect of the compressive strength on the outcome is small in comparison with other varying key factors under study. There exists some discrepancy in the literature on whether to normalize with respect to square root or cube root of concrete compressive strength [31]. Therefore, a preliminary statistical analysis is carried out for the selection of the appropriate option. Truly, Figure 1a suggests that the slope of the dataset trendline with respect to the square root of the concrete compressive strength is low; showing the elimination of concrete compressive strength from the shear strength parametric study. The data in Figure 1 are derived from the experimental published data supplementary materials. This input data are also shown in the form correlation matrix in Figure 2. The following can be observed from the plots in Figure 1:

1. By increasing the reinforcement ratio ρ, the normalized shear capacity $v_{u}/\sqrt{f_{c}^{\prime }}$ increases, Figure 1b. This is because the higher reinforcement ratio ρ ensures enhance dowel action [31,32,33].
2. By increasing the effective depth d of the beam, the normalized shear capacity decreases because of the size effect of the beam, Figure 1c [34,35,36,37,38,39].
3. By decreasing the shear span to depth ratio $\frac{a}{d}$ of the beam, the normalized shear capacity decreases because of the similar size effect of the beam, Figure 1d [40,41,42].
4. By increasing the fiber volume fraction $V_{f}$*_,_* the normalized shear capacity is increased, as indicated in Figure 1e. The shear strength of steel fiber reinforced concrete (SFRC) beam is not largely influenced by the fiber aspect ratio alone; however, this factor has a great influence when combined with the volume of fibers [1]. In this context, Narayanan and Darwish [2] originally combined the fiber aspect ratio with the fiber volume in a simple form
  (1) $$F=\left(\frac{l_{f}}{d_{f}}\right)\times V_{f}\times D_{f}$$
  where $D_{f}$ represents bond factor, which is related to the typology of the steel fibers. These authors concluded that, although the amount of increase is not quantifiable, the steel fiber reinforced concrete (SFRC) beam does increase with the increase in steel fiber volume, regardless of the concrete strength and the fiber aspect ratio.

1. By increasing the fiber’s mechanical and geometric properties, such as increasing the length of fiber or the tensile strength $\left(f_{ytent}\right)$ of the fiber, the shear strength of steel fiber reinforced concrete beams (SFRC) increases. This is because the crack control and the energy dissipation of the beam are improved as these parameters are increased, shown in Figure 1f,g [43].
2. The increase in the diameter of the fiber results in decreasing the shear strength of a steel fiber reinforced concrete (SFRC) beam as shown in Figure 1g. This is ascribed to the inverse relationship of the fiber factor (F) and the fiber diameter in Equation (1).
3. The increase in the tensile strength of the steel fiber tends to increase the shear strength of a steel fiber reinforced (SFRC) concrete beam shown in Figure 1h. It is due to the resulting increased confinement of the concrete matrix [2].

The effect of aggregate size in the steel fiber reinforced concrete (SFRC) beam is observed in Figure 1i, which is different from that of the conventional reinforced concrete beam. In the conventional RC beams, a large aggregate size tends to increase the shear strength of RC members due to the improved interlocking action. In contrast, the smaller aggregate size of steel fiber reinforced (SFRC) concrete results in a more uniform concrete matrix providing a better bond between the fiber and concrete, thereby improving the shear strength, as shown in Figure 1i [44,45].

## 5. Review of Previous Studies

Although the shear strength benefits of steel fiber reinforced concrete (SFRC) have been known for a very long time, a systematic study in this context started in the mid-1980s. Much development has taken place since, and various types of steel fiber reinforced concrete (SFRC) have been proposed based on the properties of concrete and steel fibers.

### 5.1. Experimental Investigations

Many analytical and experimental investigations have highlighted the effectiveness of steel fibers in enhancing the shear strength of steel fiber reinforced concrete (SFRC) beams, provided care is taken in the selection of these fibers. The details of the experimental results of the shear strength of steel fiber reinforced concrete (SFRC) beams are summarized in Section 5.3. These experiments are used in the current study to develop the shear strength model. The most noticeable feature of the table is that the shear strength of the steel fiber reinforced concrete (SFRC) beam increases with an increase in the volume of steel fiber. In contrast, the relation between compressive strength and shear strength tends to fluctuate. It is because the compressive strength is more related to both the fibers and concrete material properties than the fiber volume.

### 5.2. Previously Proposed Models

Based on the experimental investigation, numerous researchers have proposed prediction equations for the shear strength of steel fiber reinforced concrete (SFRC) beams. These equations are given in Table 1. It is worth noting the common characteristics of the equations. For example, most of these equations consist of the span to depth ratio and the fiber factor (F), which is related to the aspect ratio and the fiber content. Furthermore, it is noteworthy that these prediction models are followed by many design codes of practice. For example, ACI recommends using Sharma’s model [12].

### 5.3. Experimental Database

An extensive experimental database of 488 experiments of steel fiber reinforced concrete (SFRC) beams without the shear reinforcement is considered for proposing a GEP model. Moreover, only those experiments are reported in this study that have failed in shear. The experimental studies have been undertaken by several other researchers [3,4,12,13,17,24,25,29,30,46,47,49,53,54,55,56,57,58,59,60,61,62,63,64,65,66,67,68,69,70,71,72,73,74,75,76,77,78,79,80,81,82,83,84,85,86,87,88,89,90,91,92,93,94,95,96,97,98,99,100,101,102] whose data are used to develop an optimized GEP model. The data presented in Table S1 do not include the experiments performed by Keskin et al. [103], because in these experiments carbon-reinforced polymer (CFRP) specimens are used as longitudinal reinforcement. Moreover, the experiments conducted by Khan [104] are also omitted because they involve combined shear, bending, and torsional loads. The database is given in supplimetary data Tables S1 and S2. Whereas, Appendix A highlights the class boundaries of various influencing parameters in the database.

## 6. GEP Algorithm

Gene expression programming (GEP) [105] is a genetic algorithm that generates mathematical models from the supplied data in a domain-independent manner [53]. GEP is different from the genetic algorithm GAs and the genetic programming GP in terms of chromosome representation. More specifically, in GAs, the term “chromosomes” refers to linear strings of a fixed length; however, in the GPs, the term “chromosomes” refers to nonlinear entities of various sizes and shapes. Instead, GEP encompasses both a linear string with a predetermined length and a multi-dimensional ramified structure with a wide range of possible sizes and shapes.

GEP [105] selects the fittest candidates from the initial population to find the best solutions. An interesting fact about increasing gene and chromosome numbers in GEP is that they can lead to complex functions that are perfectly suited to their outcomes. Thus, there is a trade-off between achieving a simplified mathematical model, by limiting the number of genes and chromosomes, and achieving the desired level of accuracy.

Over the past decade, structural engineering has seen a significant increase in the use of GEP. Many authors have used GEP to create models for estimating structural component capacities [19,20,21]. Recently, RC beam-column joints have been successfully predicted using GEP [6], especially when the design code formulations are unavailable.

**Figure 3:**
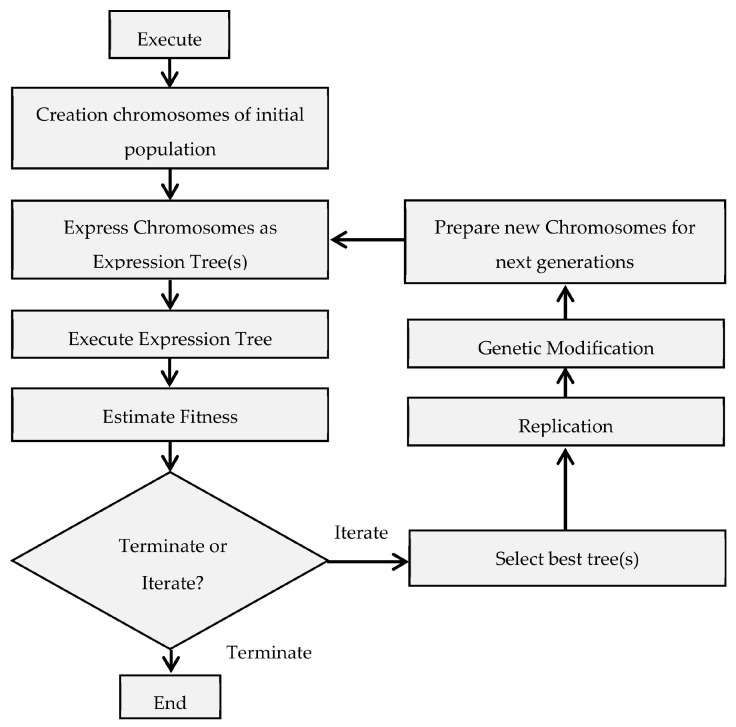
Flowchart for GEP model construct.

Figure 3 depicts the various stages of GEP optimization. The selection of control parameters, such as the function set, the terminal set, the fitness function, and the stop condition, is the first step in the optimization process. The fitness function is specified before the evolutionary algorithm is run, and this results in producing a randomly generated initial population, or what is known as ‘Chromosomes’ in the genetic programming parlance. An expression tree is constructed from these strings, and the results are compared to each chromosome’s fitness score [106]. If the fitness criterion is not met, chromosomes are selected using a roulette-wheel sampling method and then mutated to produce new generations. According to the best-fitting solution, chromosomes are decoded as the fitness function [6,7]. The newly developed GEP model along with the relevant information is discussed in the next section.

## 7. Proposed GEP Model for Estimating Steel Fiber Reinforced (SFRC) Concrete Beam

**Figure 4:**
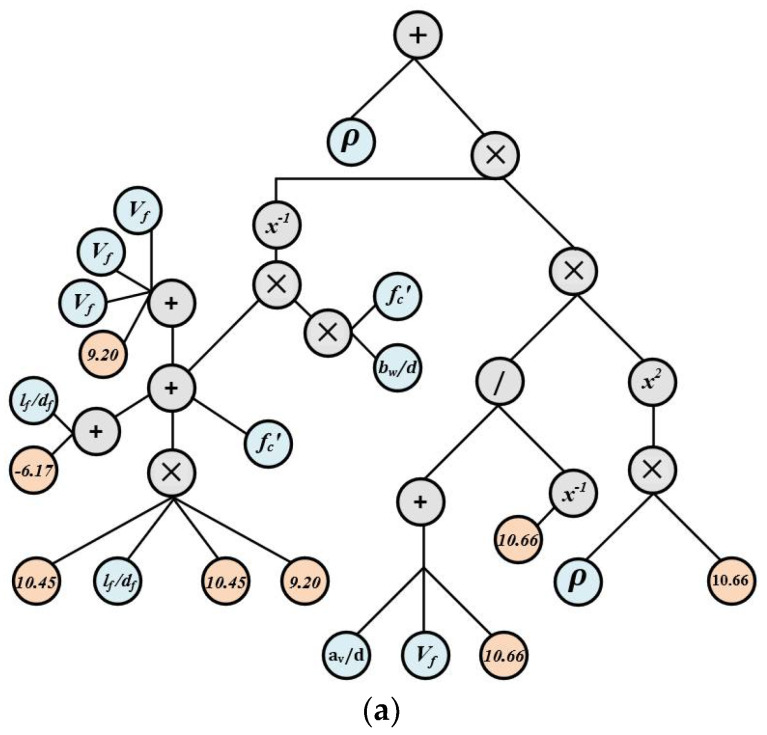

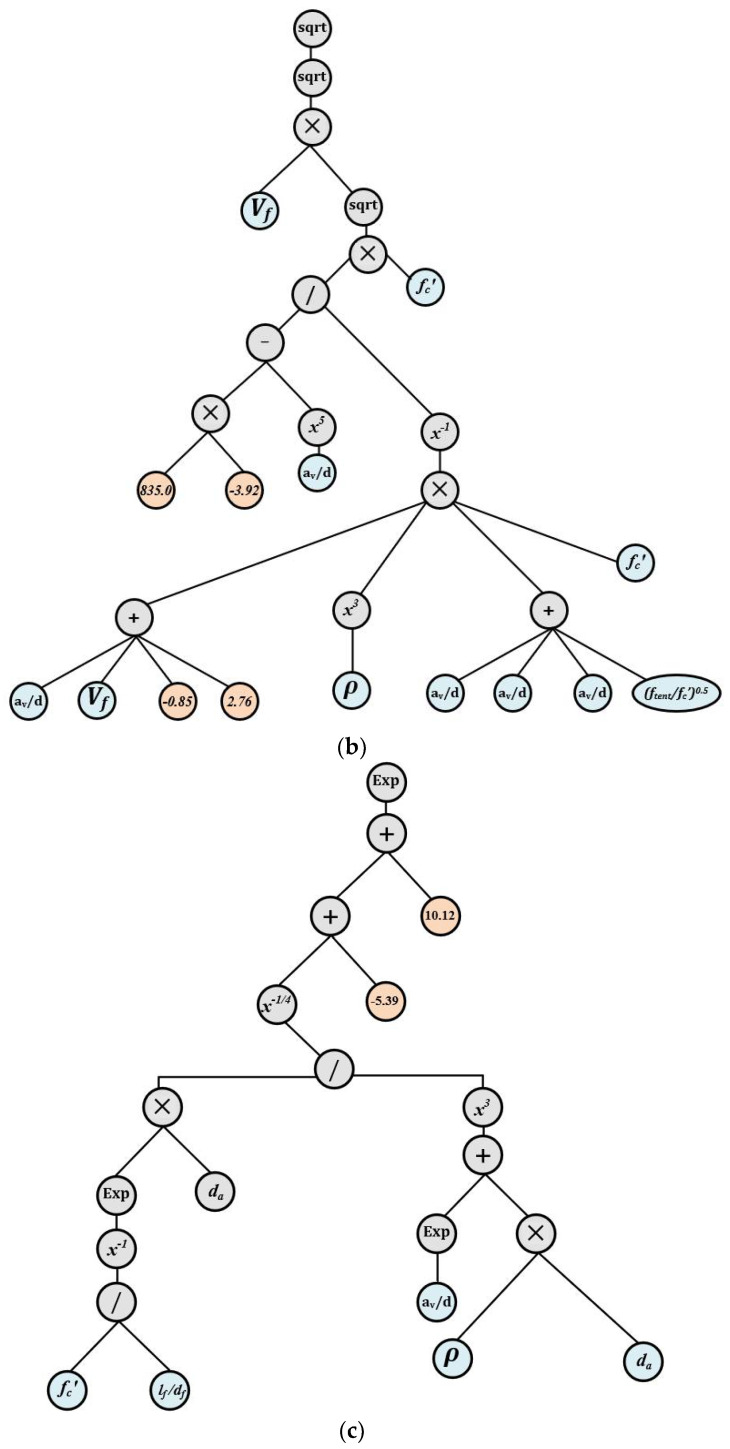
Gene Expression Trees: (**a**) Sub-ET 1, (**b**) Sub-ET 2, (**c**) Sub-ET 3.

In this section, the proposed GEP models for steel fiber reinforced (SFRC) concrete beams are discussed. The GEP [105] models generated from the previously mentioned dataset can be represented by the following simple relationships:

(2) $$v_{SFRC}=\left|v_{1SF}\times v_{2SF}\times v_{3SF}\right|\left(\mathrm{MPa}\right)$$

where

(3) $$v_{1S}=\rho +\left({\left((39.3+3V_{f}-5.17\frac{l_{f}}{d_{f}}+f_{c})\times \left(f_{c}\frac{b}{d}\right)\right)}^{-1}\times \left(\left(\frac{d_{a}+V_{f}+10.66}{{\left(10.66\right)}^{-1}}\right)*\;{\left(\;10.45\rho \right)}^{2}\right)\right)\;$$

(4) $$v_{2S}={\left({\left(\frac{\;{\left(-11.80\left(\frac{a_{v}}{d}-0.85+V_{f}+2.76\right)\times \left(3\frac{a_{v}}{d}+\frac{\sqrt{f_{ytent}}}{\sqrt{f_{c}^{\prime }}}\right)\times \left(\rho^{3}\right)\right)}^{-1}}{-3323.61-{\left(\frac{a_{v}}{d}\right)}^{5}\;}\times f_{c}^{\prime }\;\right)}^{\frac{1}{2}}\times V_{f}\;\right)}^{\frac{1}{4}}\;$$

(5) $$v_{3S}=Exp\left({\left(\frac{Exp{\left(\frac{l_{f}}{f_{c}\;d_{f}}\right)}^{-1}\times d_{a}}{{\left(\left(\rho \times d_{a}\right)+Exp\left(\frac{a_{v}}{d}\right)\right)}^{3}}\right)}^{\frac{1}{4}}+4.74\right)\;$$

(6) $$V_{SFRC}=v_{jh}b_{w}d\;\left(N\right)\;$$

where $b_{w},\;d,\;\rho ,\frac{a_{v}}{d},\;d_{a},f_{c},V_{f}\;and\;f_{ytent}$ define the width of a beam, the effective depth of the beam, the reinforcement ratio, the length to span ratio, the diameter of aggregate, the concrete compressive strength, the fiber volume fraction, and the fiber tensile strength. The gene expression trees based on Equations (3)–(5) are shown in Figure 4, along with the model construction parameters shown in Table 2.

## 8. Accuracy of the Proposed Model

**Figure 5:**
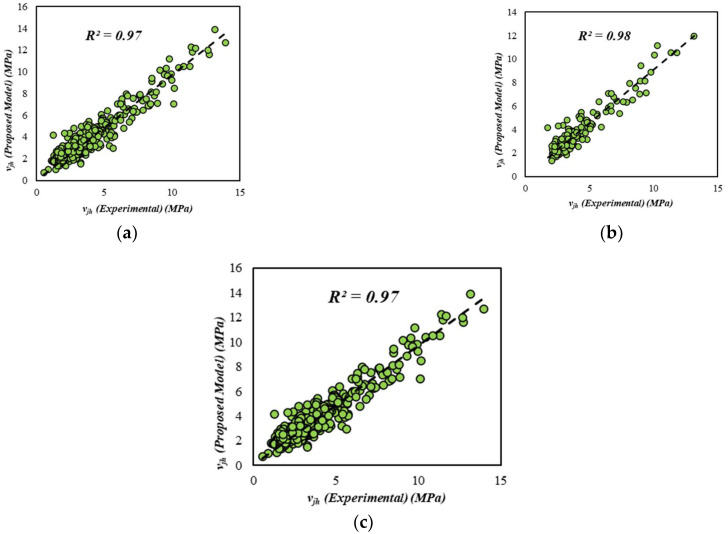
Comparison of estimated and experimental shear strength: (**a**) Training data, (**b**) Validation data, (**c**) All data.

The accuracy of any empirical model is strongly dependent on training and validation sets. It has been shown by several researchers [107,108] that a 60:40% partition gives a better result as compared to other distributions, such as 70:30%. Hence, 60 percent of the designated steel fiber reinforced (SFRC) concrete beam experiments are used as training data, while the remaining 40 percent of each category is used for validation purposes. To gain a quantitative understanding of the model, several statistical precision evaluators are used, such as the performance factor (PF), the coefficient of variation (CoV), and the average absolute error (AAE). Statistical variables such as the mean, the standard deviation, and the coefficient of variation can be used to compare the proposed model and the experimental results. It can be seen in Figure 5 that the performance factor of training, validation, and overall data for both the reinforced and unreinforced model is close to 1, which indicates an efficient model. The most accepted standardized measure for testing the precision of any model is the coefficient of variation (CoV), i.e.,

(7) $$\mathrm{CoV}\;\left(\%\right)=\left\{\frac{\mathrm{Standard}\;\mathrm{Devation}\;\left(\sigma \right)}{\mathrm{Mean}\;\left(\mu \right)}\right\}\text{×}100$$

where a lower value of the CoV measures less dispersion of the results data.

Another measure is the average absolute error (AAE), expressing the arithmetic average between the model and the experimental observations. The AAE is

(8) $$\mathrm{AAE}\;\left(\%\right)=\frac{1}{n}\sum \left[\frac{\left|V_{\mathrm{jh}}^{\mathrm{Exp}}-V_{\mathrm{jh}}^{\mathrm{Est}}\right|}{V_{\mathrm{jh}}^{\mathrm{Exp}}}\right]$$

where n is the number of test specimens.

A commonly used measure to test model reliability is the coefficient of determination ($R^{2}$) expressed as

(9) $$R^{2}=1-\frac{\sum {\left[V_{\mathrm{jh}}^{\mathrm{Exp}}-V_{\mathrm{jh}}^{\mathrm{Est}}\right]}^{2}}{\sum {\left[V_{\mathrm{jh}}^{\mathrm{Exp}}-V_{{\mathrm{jh}}_{\left(\mathrm{Mean}\right)}}^{\mathrm{Exp}}\right]}^{2}}$$

where the value of $R^{2}$ close to 1 is considered an accurate prediction.

In the same spirit, for the shear strength GEP model, the coefficient of determination is shown in Figure 5. It can be observed from the figure that the coefficient of determination for the steel fiber reinforced (SFRC) concrete shear strength model is 0.97 for training, 0.98 for validation data, and 0.97 for overall data.

**Figure 6:**
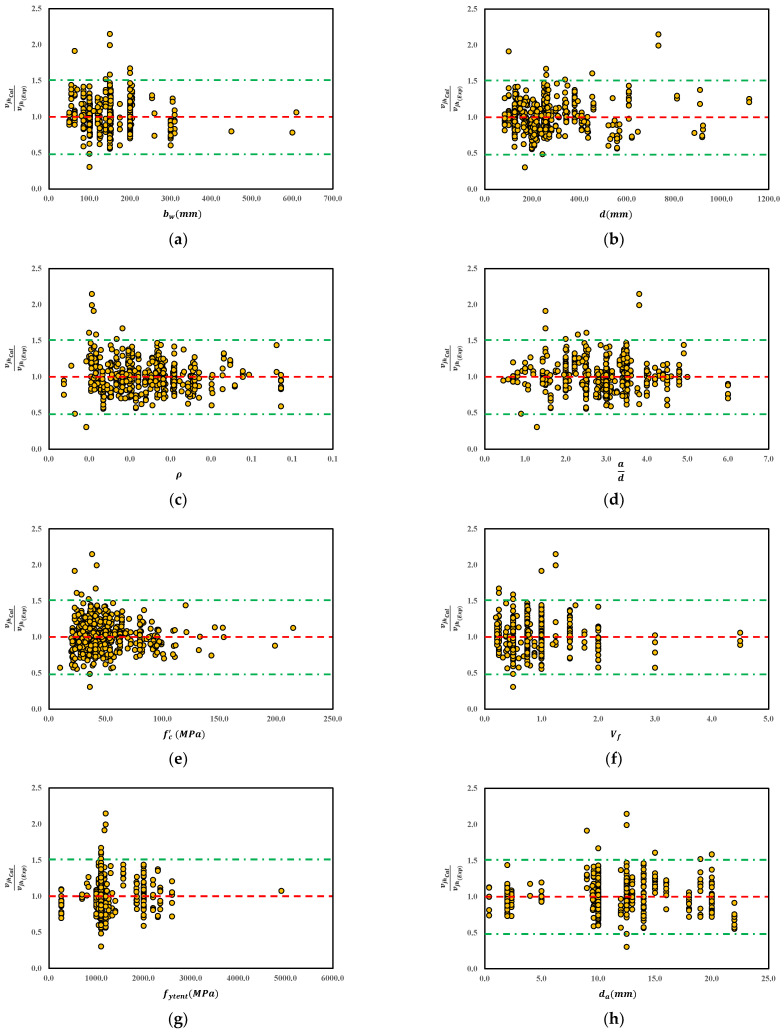
The predictive performance of the proposed steel fiber reinforced (steel fiber reinforced concrete (SFRC)) concrete beam shear strength model based on the main parameters. (**a**) Beam Width (**b**) Beam depth (**c**) Longitudnal Reinforcement (**d**) Span-to-Depth Ratio (**e**) Concrete Copmressive Strength (**f**) Fiber Volume (**g**) Fiber Tensile Strength (**h**) Diameter of Aggregate.

As a continuation of the parametric study discussed in Section 4, the sensitivity of various influencing parameters in the proposed model is investigated in Figure 6. The main influencing parameters contributing toward the shear strength of steel fiber reinforced concrete (SFRC) beams include beam width ($b_{w})$, the beam effective depth (d), the longitudinal reinforcement (ρ), the shear span to depth ratio $\left(\frac{a}{d}\right)$, the concrete compressive strength $\left(f_{c}^{\prime }\right)$, the fiber volume $\left(V_{f}\right)$, the fiber tensile strength $\left(f_{ytent}\right)$ and the diameter of aggregate $\left(d_{a}\right)$. As shown in Figure 6a, the model accuracy of beam width $b_{w}$ has an average of 1.00, which shows a suitable performance of the proposed model in various levels of $b_{w}$, since the appropriate range is within 0.5–1.5. Similarly, Figure 6b shows an identical predictive performance of 1.0 by comparing the results of the shear strength obtained analytically to experimental effective depth d of the steel fiber reinforced (steel fiber reinforced concrete (SFRC)) concrete beams. Likewise, it is of interest to note that the other influencing parameters shown in Figure 6c–f, i.e., ρ, $\frac{a}{d}$, $f_{c}^{\prime }$, $V_{f}$, $f_{ytent}$, and $d_{a}$, all exhibit an acceptable accuracy of 1.00, which lies within the appropriate range. This confirms the suitability of the proposed model to predict the experimental counterparts with reasonable accuracy.

Finally, the contribution of the variables affecting the shear strength of steel fiber-reinforced concrete beam is evaluated through a sensitivity analysis. In the view of (7) and (8), the approach proposed by Gandomi et al. [72] is employed to obtain the frequencies of the input parameters. The sensitivity ($S_{i}$) of the influencing factors is obtained by using the following equations:

(10) $$N_{i}=f_{max}\left(x_{i}\right)-f_{min}\left(x_{i}\right)$$

(11) $$S_{i}=\frac{N_{i}}{\sum_{j=1}^{n}N_{j}}\times 100$$

The $f_{max}\;\left(xi\right)$ and $f_{min}$(*xi*) are the max and min of the estimated output over the $i_{th}$. output.

## 9. Results and Discussions

**Figure 7:**
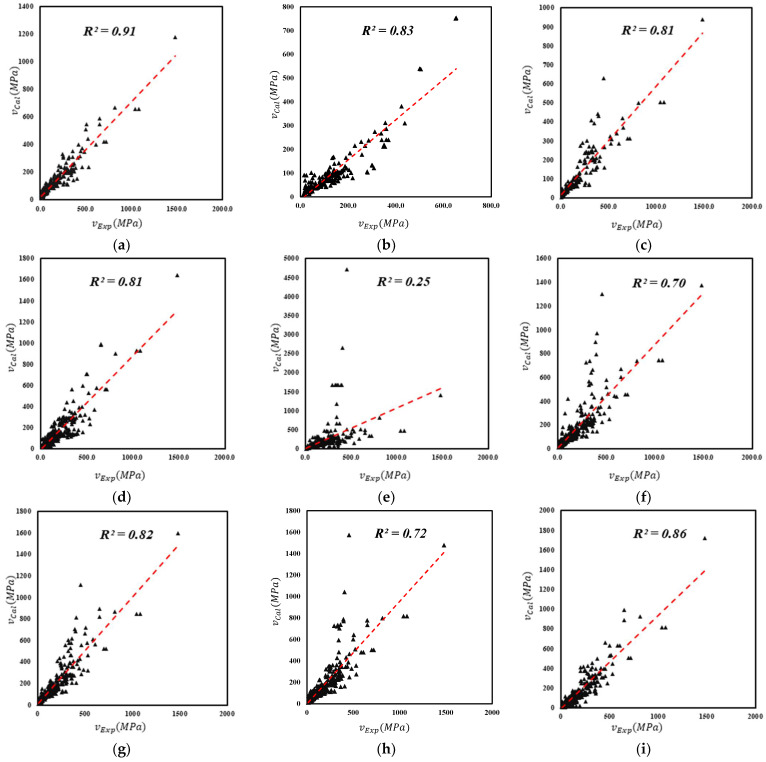

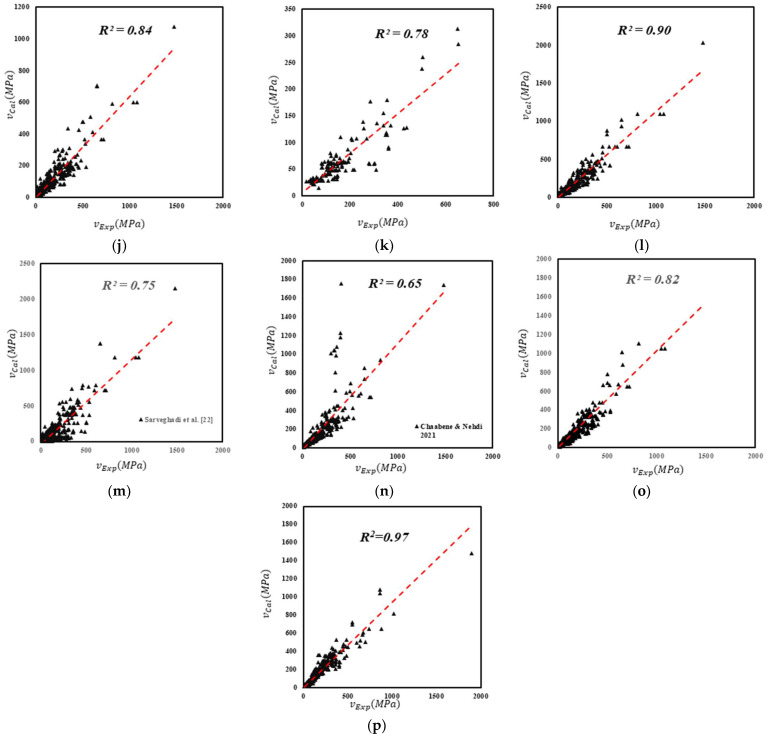
Comparison of proposed model with existing models: (**a**) Greenough and Nehdi [17], (**b**) Kunita et al. [27], (**c**) Shin et al. [46], (**d**) Sharma et al. [12], (**e**) Imam et al. [47], (**f**) Ashour et al. [48], (**g**) Kwak et al. [13], (**h**) Narayanan and Darwish [49], (**i**) Shahnewaz and Alam [18], (**j**) Shahnewaz amd Alam [51], (**k**) RILEM [52], (**l**) Gandomi et al [50], (**m**) Sarveghadi et al [16], (**n**) Chaabene and Nehdi 2021 [53], (**o**) Sabetifar et al. [22], (**p**) Proposed model.

The graphical plots of experimental and predicted values are demonstrated herein. It can be seen from Figure 7 that the coefficient of determination of the current model is R^2^ = 0.97, which shows better prediction when compared to the other available models. In addition, higher performance factor and lower average absolute error suggest the merit of the current model. The model of Imam et al. [47] demonstrates the least predictive ability because the model is developed using a narrower range of aggregate sizes. Therefore, this model fails to predict the shear strength accurately where the aggregate size does not fall within the range of validity.

**Figure 8:**
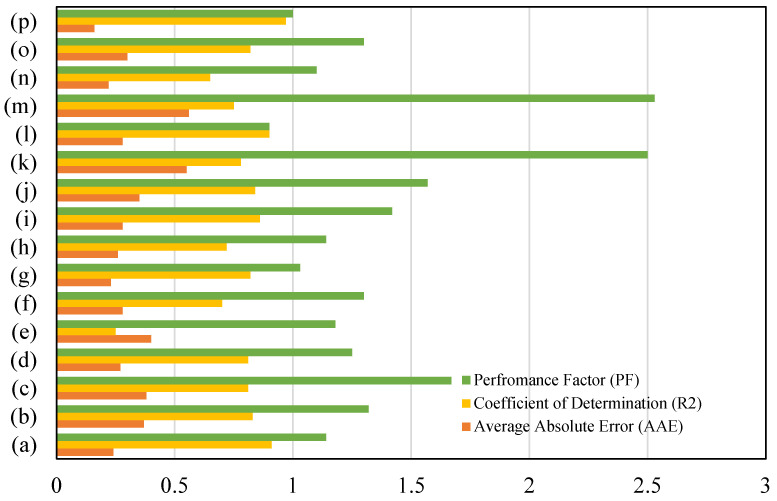
(**a**) Greenough and Nehdi [17], (**b**) Kunita et al. [27], (**c**) Shin et al. [46], (**d**) Sharma et al. [12], (**e**) Imam et al. [47], (**f**) Ashour et al. [48], (**g**) Kwak et al. [13], (**h**) Narayanan and Darwish [49], (**i**) Shahnewaz and Alam [18], (**j**) Shahnewaz amd Alam [51], (**k**) RILEM [52], (**l**) Gandomi et al [50], (**m**) Sarveghadi et al [16], (**n**) Chaabene and Nehdi 2021 [53], (**o**) Sabetifar et al. [22], (**p**) Proposed model.

The Chaabene and Nehdi model [53] has one of the lowest predictive abilities in the list in Table 3 and Figure 8, as its coefficient of determination is 0.65 and the average absolute error is 22%. This model has been validated on 2000 synthetic data points. It is anticipated that the use of synthetic data points does not improve the model performance. The performance factor of the Chaabene and Nehdi [53] model is 1.10, which is very close to the benchmark statistical value of 1.

The remaining models reflect a greater difference in the predictive performance, see Table 3. This is either due to the employment of very few data points for establishing the empirical model, or omission of important influencing parameters. For example, Sharma [12] proposed a model for the shear capacity of shallow beams reinforced with hook-type fibers. The exclusion of key parameters, such as the fiber aspect ratio and fiber reinforcement ratio, leads to reduced reliability of shear strength prediction of Sharma’s model. This clearly shows that these key factors have a substantial influence on the shear strength of steel fiber reinforced concrete (SFRC) beams. Therefore, in the context of the currently established database, Sharma’s model, which is also adopted by ACI, shows more variation for beams with span to depth ratio $\frac{a}{d}<3$ than beams with $\frac{a}{d}>3$. On the contrary, the current genetic model, which is created on a larger database and encompasses several key influencing parameters, gives an accurate prediction of the shear strength of steel fiber reinforced concrete (SFRC) beams.

**Figure 9:**
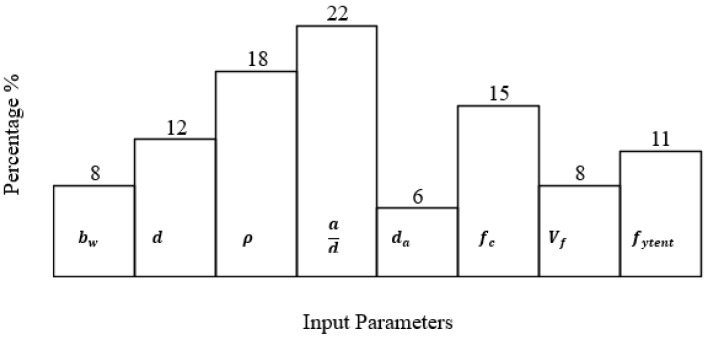
Contribution of input parameters in the proposed GEP model.

Sensitivity analysis is carried out to assess the contribution of each input variable to the predicted value. The frequency values of these variables are presented in Figure 9. It is clear from this figure that the proposed model is most sensitive to the shear span to depth ratio $\left(\frac{a}{d}\right)$ and least sensitive to the diameter of aggregate $\left(d_{a}\right)$. The frequency value of the fiber tensile strength $\left(f_{ytent}\right)$ is 11%, indicating a sizable sensitivity. The sensitivity of different input parameters is in accordance with the parametric study of Section 4.

## 10. Conclusions

The present study is aimed at developing an efficient genetic shear capacity model of steel fiber reinforced concrete (SFRC) beams in the absence of stirrups. In contrast to the available shear capacity models, the important influencing parameter of fiber tensile strength is implemented in the current model resulting in higher accuracy. A total number of 488 experiments are employed to estimate the shear strength of steel fiber reinforced concrete (SFRC) beams, out of which 190 experiments are used to validate the proposed model. The proposed genetic model is benchmarked against 14 previous empirical or semi-analytical models. The following conclusions are drawn:

- It is observed that an increase in the concrete compressive strength, reinforcement ratio, fiber volume, and fiber tensile strength, each increases the shear strength of steel fiber reinforced concrete (SFRC) beams. Contrarily, an increase in the span to depth ratio, the beam effective depth, and the aggregate diameter, each result in a decrease in the shear strength of the beam.
- The proposed empirical equation can accurately determine the shear strength of steel fiber reinforced concrete (SFRC) beams by incorporating all the aforementioned influencing factors. Moreover, the behavior of different influencing parameters in the proposed model shows consistency with the experimental data.
- Statistical investigation demonstrates the accuracy and validity of the proposed model, as the values obtained from the statistical analysis are very close to the benchmark values. More specifically, 0.97 of coefficient of determination (R2) is close to the benchmark value of 1.00, which indicates the higher reliability of the proposed model. Similarly, the performance factor of the proposed model is 1.00, which is also the benchmark value. The average absolute error (AAE) of the model is 16%, which firmly confirms the accuracy of the proposed model.
- A particular virtue of the proposed model is that it can approximate the shear capacity of the steel fiber reinforced concrete beam more closely than other available models. A comparison is made with fourteen different models to evidence the predictive ability of the developed model.
- In conclusion, it is believed that the proposed regression model offers a robust predictive apparatus for determining the shear capacity of steel fiber reinforced concrete (SFRC) beams. Hence, the model can be confidently prescribed for the shear design of these beams.
- In future work, it is planned to conduct experimental and numerical studies investigating the effect of steel fiber strength on the shear capacity of steel fiber reinforced concrete (SFRC) beams. This can also be an effective way to further validate the current GEP model.

## Figures and Tables

**Table 1:** Available shear strength models of steel fiber reinforced (steel fiber reinforced concrete (SFRC)) concrete beams.

Author	Proposed Equation	Stirrups (Y/N)	Equation Description	Limitations
Greenoughand et al. [17]	$v_{u}=0.35\left(1+\sqrt{\frac{400}{d}}\right){\left(f_{c}^{\prime }\right)}^{0.18}{\left((1+F)\rho \frac{d}{a}\right)}^{0.4}+0.9\eta_{o}\tau F$	No	Genetic Programming	$\frac{a}{d}\geq$2.5
Khuntia et al. [27]	$\upsilon_{u}=\left(0.167\left(2.5*\frac{d}{a}\right)\right)\sqrt{f_{c}^{\prime }}$	No	ACI Code Modification	$\frac{a}{d}<$ 2.5 0.25≤ρ≤ 2.5 $20\leq f_{c}^{\prime }\leq$ 100
	$\upsilon_{u}=0.35\left(0.167+0.25F\right)\sqrt{f_{c}^{\prime }}$	No	ACI Code Modification	$\frac{a}{d}\geq$2.5 0.25≤ρ≤ 2.0 $20\leq f_{c}^{\prime }\leq$ 100
Shin et al. [46]	$\upsilon_{u}=0.22f_{SP}+217\rho \frac{d}{a}+0.834\left(0.41\tau F\right)$	No		$HSC\;\frac{a}{d}<$ 3
	$\upsilon_{u}=0.19f_{SP}+93\rho \frac{d}{a}+0.834\left(0.41\tau F\right)$	No		$\frac{a}{d}\geq \;$3, HSC
Sharma [12]	$\upsilon_{u}=\frac{2}{3}f_{t}{\left(\frac{d}{a}\right)}^{\frac{1}{4}}$ $\;\text{where},\;f_{t}\approx 9.5\left(\sqrt{f_{c}^{\prime }}\right)psi$	No		
Imam et al. [47]	$\upsilon_{u}=0.7\left(\frac{1}{\sqrt{\left(1+\frac{d}{d_{a}}\right)}}\right)\sqrt[{3}]{\rho }\left(f_{c}^{i0.44}\left(1+F^{0.33}\right)870\sqrt{\frac{\rho }{{\left(a/d\right)}^{5}}}\right)$	No	Regression Analysis	Require max. aggregate size
	$\upsilon_{u}=0.7\left(\frac{1}{\sqrt{\left(1+\frac{d}{25d_{a}}\right)}}\right)\sqrt[{3}]{\rho }\left(f_{c}^{i0.44}\left(1+F^{0.33}\right)+870\sqrt{\frac{\rho }{{\left(a/d\right)}^{5}}}\right)$	No	Regression Analysis	Requires max aggregate size
Ashour et al. [48]	$\upsilon_{u}=\left(\frac{2.5}{a/d}\right)+0.41\tau F\left(2.5-\frac{a}{d}\right)$ $\upsilon_{u}=\left(0.7\sqrt{f_{c}^{\prime }}+7F\right)\frac{d}{a}+17.2\rho \frac{d}{a}$	No	ACI Code Modification	$\frac{a}{d}<$2.5 More accurate with HSC
	$\upsilon_{u}=\left(2.11\sqrt[{3}]{f_{c}^{\prime }}+7F\right){\left(\rho \frac{a}{d}\right)}^{0.33333}$ $\upsilon_{u}=\left(0.7\sqrt{f_{c}^{\prime }}+7F\right)\frac{d}{a}+17.2\rho \frac{d}{a}$	No	Regression Analysis ACI Code Modification	$\frac{a}{d}=$2.5 More accurate with HSC
Kwak et al. [13]	$\upsilon_{u}=3.7\left(3.4\frac{d}{a}{\left(\frac{f_{c}^{\prime }}{20-\sqrt{F}}+0.7+\sqrt{F}\right)}^{\frac{2}{3}}{\left(\frac{\rho d}{a}\right)}^{\frac{1}{3}}\right)+0.8\left(0.41\tau F\right)$	No		$\frac{a}{d}\leq 3.4$
	$\upsilon_{u}=3.7\left(3.4\frac{d}{a}{\left(\frac{f_{c}^{\prime }}{20-\sqrt{F}}+0.7+\sqrt{F}\right)}^{\frac{2}{3}}{\left(\frac{\rho d}{a}\right)}^{\frac{1}{3}}\right)+0.8\left(0.41\tau F\right)$	No		$\frac{a}{d}=$3.4
Narayanan and Darwish [49]	$\upsilon_{u}=2.8\frac{d}{a}\left(0.24{\left(\frac{f_{c}^{\prime }}{20-\sqrt{F}}+0.7+\sqrt{F}\right)}^{\frac{2}{3}}+80\rho \frac{d}{a}\right)+\left(0.41\tau F\right)$.	No	Regression Analysis	$\frac{a}{d}\leq 2.8$
	$\upsilon_{u}=\left(0.24{\left(\frac{f_{c}^{\prime }}{20-\sqrt{F}}+0.7+\sqrt{F}\right)}^{\frac{2}{3}}+80\rho \frac{d}{a}\right)$ +(0.41τF)	No	Regression Analysis	$\frac{a}{d}=$2.8
Gandomi et al. [50]	$v_{u}=\frac{2d}{a}\left(\rho f_{c}^{\prime }+v_{b}\right)+\frac{d}{2a}\;\frac{\rho }{{\left(288\rho -11\right)}^{4}}+2$	No	Genetic Programming	
Shahnewaz and Alam [18]	$v_{u}=3.2+0.072f_{c}^{\prime }+\rho V_{f}\left(1.26-0.25\frac{a}{d}\right)-\frac{a}{d}\;\left(1.92+0.017f_{c}^{\prime }-0.38\frac{a}{d}\right)$	No		
Sarveghadi et al. [16]	$\frac{f_{t}^{\prime }+v_{b}}{\frac{a}{d}-\rho +\frac{3\rho }{V_{b}}\left(V_{b}+2+\frac{a}{d}+f_{t}+4\rho f_{t}\right)}+V_{b}$	No		$f_{c}^{\prime }<41.4\;MPa$ $f_{t}^{\prime }=0.79\sqrt{f_{c}^{\prime }}$
	$\frac{d}{a}\left(\;2f_{t}^{\prime }+\frac{\left(\frac{a}{d}\right)}{\rho +\rho \left(4+V_{b}\right)\left(\frac{a}{d}+\rho \right)\left(-1-\frac{a}{d}\right)}-2\right)+V_{b}$	No		$f_{c}^{\prime }>41.4\;MPa$ $f_{t}^{\prime }=0.79\sqrt{f_{c}^{\prime }}$
Shahnewaz and Alam [51]	$v_{u}=0.2+0.034f_{c}^{\prime }+19\rho^{0.087}-5.8{\left(\frac{a}{d}\right)}^{\frac{1}{2}}+3.4V_{f}-800{\left(\frac{l_{f}}{d_{f}}\right)}^{-1.6}-12{\left(\left(\frac{a}{d}\right)V_{f}\right)}^{0.05}-197{\left(\left(\frac{a}{d}\right)\left(\frac{l_{f}}{d_{f}}\right)\right)}^{-1.4}+105{\left(V_{f}\left(\frac{l_{f}}{d_{f}}\right)\right)}^{-2.12}$			$\frac{a}{d}\leq 2.8$
	$v_{u}=0.2+0.072{\left(f_{c}^{\prime }\right)}^{0.85}+12.5\rho^{0.084}-24{\left(\frac{a}{d}\right)}^{0.07}+13.5V_{f}^{0.07}+450{\left(\frac{l_{f}}{d_{f}}\right)}^{-2}-0.0002{\left(V_{f}\left(\frac{l_{f}}{d_{f}}\right)\right)}^{3.9}-27.69{\left(\left(\frac{a}{d}\right)\left(\frac{l_{f}}{d_{f}}\right)\right)}^{-0.84}+1181{\left(V_{f}\left(\frac{l_{f}}{d_{f}}\right)\right)}^{-2.69}-21.89{\left(\left(\frac{a}{d}\right)\left(\frac{l_{f}}{d_{f}}\right)V_{f}\right)}^{-0.9}$	No	Genetic Programming	$\frac{a}{d}\leq 2.8$
RILEM [52]	$v_{Rd}=v_{cd}+v_{fd}v_{cd}=0.12{\left(100\rho f_{c}^{\prime }\right)}^{\frac{1}{3}}\;$ $k=1+\sqrt{\frac{200}{d}}\leq 2;\rho \leq 2\%\;V_{fd}$ $=k_{f}k_{1}\tau_{fd}:k_{1}=1+\sqrt{\frac{200}{d}}\leq 2\;;k_{f}=1$ $\tau_{fd}=0.12f_{Rk,4}$$;\;f_{Rk,4}=1\;\mathrm{MPa}$ assuming sufficient fiber dosage	No		
Chaabene and Nehdi [53]	$v_{u}=0.921+\frac{0.694\times \ln\left(1.786F+1.091\rho \right)\sqrt{0.787f_{c}^{\prime }+\frac{4.863\rho \sqrt{0.798f_{c}^{\prime }}}{{\left(\frac{a}{d}\right)}^{3}}}}{\left(\frac{a}{d}\right)}$.	No	Genetic Programming	
Sabetifar et al. [22]	$v_{u}=F+2\rho +\sqrt{\frac{\rho f_{c}^{\prime }{\left(F+3.58\right)}^{2}}{\left(\frac{a}{d}\right)}}-\rho^{2}\left(f_{c}^{\prime }+8.52\right)+F\left(F-0.73\right)(\rho^{\rho }-\sqrt{\left(\frac{a}{d}\right)}$	No	Genetic Programming	
$F=V_{f}\frac{l_{f}}{d_{f}}D_{f}$ $\alpha =1\;N/mm^{2}$ $\tau =4.15\;N/mm^{2}$				

**Table 2:** Model Construction Parameter.

Function Set	Exp,Ln, +, −, /, *, sqrt, ×2,3,4,5
Chromosomes	130
Head size	16
Linking function	Multiplication
Number of genes	3
Mutation rate	0.0014
Inversion rate	0.1
One-point recombination rate	0.0027
Two-point recombination rate	0.0027
Gene recombination rate	0.0027
Gene transposition rate	0.0027
Constants per gene	10
Lower/Upper bound of constants	−20/20

**Table 3:** Predictive capability of shear strength models of steel fiber reinforced (SFRC) concrete beam.

Author	$PF=\frac{v_{jh}^{\;\;Exp}}{v_{jh}^{\;\;Est}}$	AAE (%)	$R^{2}$
	Mean		
Greenough and Nehdi [17]	1.14	24.00	0.91
Kunita et al. [27]	1.32	37.00	0.83
Shin et al. [46]	1.67	38.00	0.81
Sharma et al. [12]	1.25	27.00	0.81
Imam et al. [47]	1.18	40.00	0.25
Ashour et al. [48]	1.30	28.00	0.70
Kwak et al. [13]	1.03	23.00	0.82
Narayanan and Darwish [49]	1.14	26.00	0.72
Shahnewaz and Alam [18]	1.42	28.00	0.86
Shahnewaz and Alam [51]	1.57	35.00	0.84
RILEM [52]	2.50	55.00	0.78
Gandomi et al. [50]	0.90	28.00	0.90
Sarveghadi et al. [16]	2.53	56.00	0.75
Chaabene and Nehdi [53]	1.10	22.00	0.65
Sabetifar et al. [22]	1.30	30.00	0.82
**Proposed Model**	**1.00**	**16.00**	**0.97**

**Figure A1:**
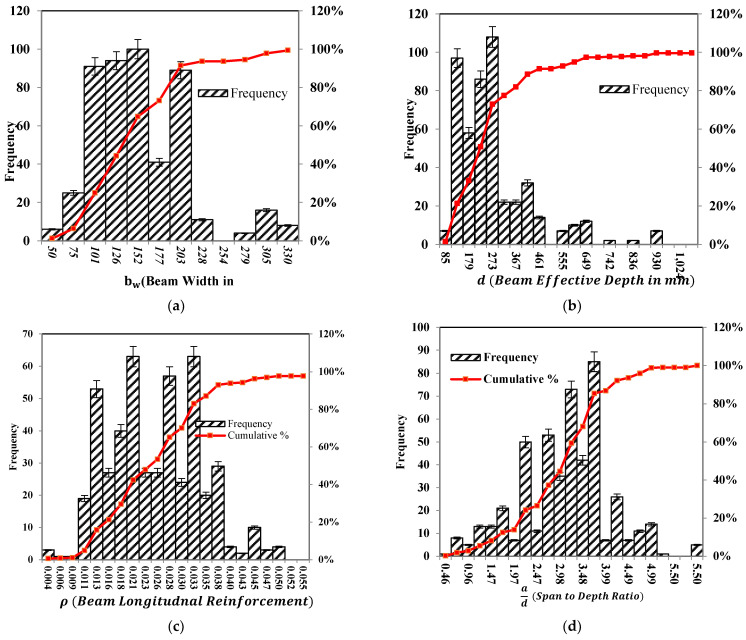

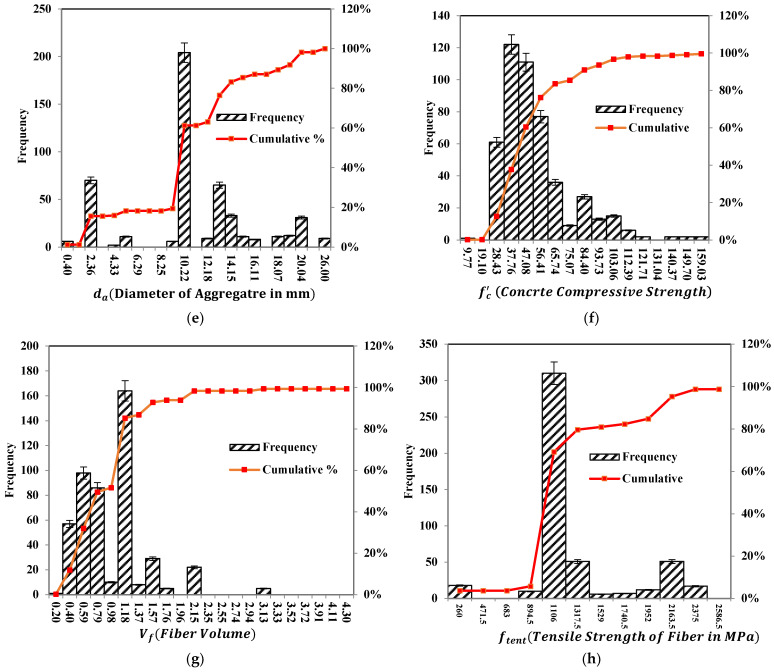
Range of different parameters in dataset: (**a**) Width of beam, (**b**) Effective depth of beam, (**c**) Reinforcement Ratio, (**d**) Length to span ratio, (**e**) Diameter of aggregate, (**f**) Concrete strength, (**g**) Fiber volume fraction, (**h**) Fiber tensile strength.

## Data Availability

The authors confirm that the data supporting the findings of this study are available within the article in Supplementary Materials Tables S1 and S2. Additionally, the papers are cited in Section 5.3 of the manuscript.

## Supplementary Materials

The following supporting information can be downloaded at: https://www.mdpi.com/article/10.3390/ma15113758/s1, Table S1: Properties of steel fiber reinforced (steel fiber reinforced concrete (SFRC)) concrete beam; Table S2: Properties of Steel Fiber.

## Appendix A

### Range of Different Influencing Parameters in the Database

After identifying and grouping various influencing parameters in the database, the next stage is recognizing various parameters constituting a group. Figure A1 entails the class boundaries of various groups based on the shape of the steel fibers. It is noteworthy that the maximum tested height of the beam is 1220 mm. The database comprises various fiber types. The following observations are made:

- Examining the concrete compressive strength ($f_{c}^{\prime })$ in Figure A1a, the results lie within the range of normal strength concrete, with some outlying observations that are due to high and ultra-high-strength concrete.
- The bar chart plot of the effective depth (d) shows crowding in a small effective depth range.
- The amount of longitudinal steel (ρ) is large in all the specimens in the database, thus compelling beam failure in shear mode rather than flexure. Figure 1c shows the range of values of the longitudinal steel from 1.5–3.5%.
- From Figure A1d, it can be seen that the shear span to depth ratio with $\frac{a}{d}$ = 3.5 is the most frequently used value.
- The fiber volume fraction $\left(V_{f}\right)$. shows crowding in the range of 0.5–1.5%.
- The tensile strength of steel fiber $\left(f_{\mathrm{ytent}}\right)$ exists within a large range, i.e., 260–2587. This wide range of influencing factors data is used to establish an accurate model to estimate the shear strength of steel fiber reinforced concrete (SFRC) beam.

## Appendix B

**List of Acronyams**	
d.	Effective depth of beam (mm)
$b_{w}$.	Width of beam (mm)
ρ	Beam longitudinal reinforcement
$f_{c}^{\prime }$	Concrete compressive strength (MPa)
F	Fiber factor
$\eta_{o}$	fiber orientation factor
τ.	Shear stress in beam (MPa)
a	Length of beam (mm)
$a_{v}$	Shear Length of beam (mm)
$f_{SP}$	Split cylinder test of beam (MPa)
$f_{t}$	Concrete tensile strength (MPa)
$d_{a}$	Shear depth of beam (mm)
$V_{f}$	Fiber Volume
$f_{t}^{\prime }$	Splitting tensile strength of concrete ($0.79\sqrt{f_{c}^{\prime }})$ (MPa)
$V_{b}$	Shear force in beam (MPa)
$l_{f}$	Length of fiber (mm)
$d_{f}$	Diameter of fiber (mm)
$v_{cd}$	Member shear resistance without shear reinforcement (MPa)
k	Constant (no other information is given in the paper)
$k_{f}$	Flange contribution factor of a T-section
$k_{1}$	Constant (no other information is given in the paper)
$\tau_{fd}$	Design value of the increase in shear strength due to steel fibers (MPa)
$f_{Rk,4}$	Factor used for accounting fiber dosage
$f_{tent}$	Tensile strength of fiber (MPa)
$d_{a}$	Diameter of aggregate (mm)
$D_{f}$	Bond factor
$V^{Est}$	Estimated steel fiber reinforced concrete beam shear capacity (MPa)
$V^{Exp}$	Experimental steel fiber reinforced concrete (SFRC) beam shear capacity (MPa)
