# Supplementary material for: Improved Shear Strength Prediction Model of Steel Fiber Reinforced Concrete Beams by Adopting Gene Expression Programming
Source: Materials (Basel). 2022 May 24;15(11):3758. doi: 10.3390/ma15113758 (PMC9181210; doi:10.3390/ma15113758)
Supplement: Supplementary file 1 [file materials-15-03758-s001.zip › materials-1623194-supplementary.pdf]

Table S1: Properties of SFRC beam

Reference	ID	b_w	h	d	l_{span}	ρ	a/d	a_v/d	d_a	$f_{c,cyl}$	Fiber Type	V_f
		(mm)	(mm)	(mm)	(mm)	(-)	(-)	(-)	(mm)	(MPa)		(%)
Singh & Jain 2014	*D-I*	150	300	251	1470	0.0267	3.49	3.09	12.5	28.1	*hooked*	0.75
	D-II	150	300	251	1470	0.0267	3.49	3.09	12.5	25.3	*hooked*	0.75
	E-I	150	300	251	1470	0.0267	3.49	3.09	12.5	27.9	*hooked*	1
	E-II	150	300	251	1470	0.0267	3.49	3.09	12.5	26.2	*hooked*	1
	F-I	150	300	251	1470	0.0267	3.49	3.09	12.5	28.1	*hooked*	1.5
	F-II	150	300	251	1470	0.0267	3.49	3.09	12.5	27.3	*hooked*	1.5
	G-I	150	300	251	1470	0.0267	3.49	3.09	12.5	27.5	*hooked*	0.5
	G-II	150	300	251	1470	0.0267	3.49	3.09	12.5	24.9	*hooked*	0.5
	H-I	150	300	251	1470	0.0267	3.49	3.09	12.5	27.8	*hooked*	0.75
	H-II	150	300	251	1470	0.0267	3.49	3.09	12.5	27.3	*hooked*	0.75
	I-I	150	300	251	1470	0.0267	3.49	3.09	12.5	26.3	*hooked*	1
	I-II	150	300	251	1470	0.0267	3.49	3.09	12.5	27.1	*hooked*	1
	K-I	150	300	251	1470	0.0267	3.49	3.09	12.5	53.4	*hooked*	0.75
	K-II	150	300	251	1470	0.0267	3.49	3.09	12.5	54.1	*hooked*	0.75
	L-I	150	300	251	1470	0.0267	3.49	3.09	12.5	53.2	*hooked*	1
	L-II	150	300	251	1470	0.0267	3.49	3.09	12.5	55.3	*hooked*	1
	P-I	150	300	251	1470	0.0267	3.49	3.09	12.5	64.6	*hooked*	1.5
	P-II	150	300	251	1470	0.0267	3.49	3.09	12.5	59.9	*hooked*	1.5
	AA-I	150	300	251	1470	0.0267	3.49	3.09	12.5	47.8	*hooked*	0.5
	AA-II	150	300	251	1470	0.0267	3.49	3.09	12.5	49.5	*hooked*	0.5
	M-I	150	300	251	1470	0.0267	3.49	3.09	12.5	55.3	*hooked*	0.75
	M-II	150	300	251	1470	0.0267	3.49	3.09	12.5	56.4	*hooked*	0.75
	N-I	150	300	251	1470	0.0267	3.49	3.09	12.5	53.4	*hooked*	1
	N-II	150	300	251	1470	0.0267	3.49	3.09	12.5	51	*hooked*	1
	R-I	150	300	251	1470	0.0267	3.49	3.09	12.5	27.8	*crimped*	1
	R-II	150	300	251	1470	0.0267	3.49	3.09	12.5	27.2	*crimped*	1
	U-I	150	300	251	1470	0.0267	3.49	3.09	12.5	27.6	*crimped*	1
	U-II	150	300	251	1470	0.0267	3.49	3.09	12.5	27.9	*crimped*	1
	W-I	150	300	251	1470	0.0267	3.49	3.09	12.5	34.7	*crimped*	1
	W-II	150	300	251	1470	0.0267	3.49	3.09	12.5	36.2	*crimped*	1
	Z-I	150	300	251	1470	0.0267	3.49	3.09	12.5	37	*crimped*	1
	Z-II	150	300	251	1470	0.0267	3.49	3.09	12.5	38.3	*crimped*	1
Sahoo & Sharma 2014	*M-25-0.50*	150	300	261	1800	0.0116	2.30	1.92	20.0	28.7	*hooked*	0.5
	M20-S-0.75	150	300	261	1800	0.0195	3.45	3.07	20.0	32.9	*hooked*	0.75
	M20-S-1	150	300	261	1800	0.0195	3.45	3.07	20.0	23.8	*hooked*	1
	M20-S-1.25	150	300	261	1800	0.0195	3.45	3.07	20.0	24.1	*hooked*	1.25
Shoaib, Lubell and Bindiganavile 2015	*L31*	310	308	258	1548	0.0184	3.00	2.42	10.0	22	*hooked*	1
	L32	310	308	258	1548	0.0245	3.00	2.42	10.0	31	*hooked*	1
	L62	300	600	550	3300	0.0119	3.00	2.73	10.0	30	*hooked*	1
Manju et al 2017	*SH1*	140	220	175	2000	0.0128	1.50	0.93	12.0	82	*hooked*	0.5
	SH2	140	220	175	2000	0.0128	1.50	0.93	12.0	83.2	*hooked*	1
	SH3	140	220	175	2000	0.0128	1.50	0.93	12.0	83.8	*hooked*	1.5

	SH4	140	220	175	2000	0.0128	2.50	1.93	12.0	82	*hooked*	0.5
	SH5	140	220	175	2000	0.0128	2.50	1.93	12.0	83.2	*hooked*	1
	SH6	140	220	175	2000	0.0128	2.50	1.93	12.0	83.8	*hooked*	1.5
Arslan et al. 2017	*A2.5F1.0A*	150	230	200	1000	0.0134	2.50	2.00	22.0	33.68	*hooked*	1
	A2.5F1.0b	150	230	200	1000	0.0134	2.50	2.00	22.0	24.53	*hooked*	1
	A2.5F2.0	150	230	200	1000	0.0134	2.50	2.00	22.0	21.43	*hooked*	2
	A2.5F3.0	150	230	200	1000	0.0134	2.50	2.00	12.0	9.77	*hooked*	3
	A3.5F1.0	150	230	200	1400	0.0134	3.50	3.00	22.0	20.21	*hooked*	1
	A3.5F2.0	150	230	200	1400	0.0134	3.50	3.00	22.0	21.43	*hooked*	2
	A3.5F3.0	150	230	200	1400	0.0134	3.50	3.00	12.0	27.91	*hooked*	3
	A4.5F1.0	150	230	200	1800	0.0134	4.50	4.00	22.0	24.53	*hooked*	1
	A4.5F2.0	150	230	200	1800	0.0134	4.50	4.00	22.0	21.43	*hooked*	2
Parra-Montesinos et al. 2006	*11*	152	457.2	381	2766.2	0.0271	3.40	3.41	10.0	49.2	*hooked*	1
	7	152	457.2	381	2766.2	0.0271	3.40	3.41	10.0	31	*hooked*	1.5
	10	152	457.2	381	2766.2	0.0271	3.40	3.41	10.0	44.9	*hooked*	1.5
	9	152	457.2	381	2766.2	0.0271	3.40	3.41	10.0	44.9	*hooked*	1.5
	12	152	457.2	381	2766.2	0.0271	3.40	3.41	10.0	49.2	*hooked*	1
	8	152	457.2	381	2766.2	0.0271	3.40	3.41	10.0	31	*hooked*	1.5
	4	152	457.2	381	2817	0.0271	3.50	3.47	10.0	38.1	*hooked*	1
	3	152	457.2	381	2817	0.0271	3.50	3.47	10.0	38.1	*hooked*	1
	1	152	457.2	381	2817	0.0197	3.50	3.47	10.0	38.1	*hooked*	1
	2	152	457.2	381	2817	0.0197	3.50	3.47	10.0	38.1	*hooked*	1
Rosenbusch & Teutsch 2003	*2.2/2*	200	300	260	952.64	0.0181	1.50	1.51	10.0	41.2	*hooked*	0.25
	2.2/3	200	300	260	952.64	0.0181	1.50	1.51	10.0	40.3	*hooked*	0.76
	2.4/2	200	300	260	1450.48	0.0181	2.50	2.46	10.0	40	*hooked*	0.25
	2.4/3	200	300	260	1450.48	0.0181	2.50	2.46	10.0	38.7	*hooked*	0.76
	2.3/2	200	300	260	1450.48	0.0115	2.50	2.46	10.0	40	*hooked*	0.25
	2.3/3	200	300	260	1450.48	0.0115	2.50	2.46	10.0	38.7	*hooked*	0.76
	*T15*100-SFRC -2*	200	500	460	3248.8	0.0280	3.40	3.35	10.0	37.7	*hooked*	0.5
	*T23*50-SFRC -2*	200	500	460	3248.8	0.0280	3.40	3.35	10.0	38.8	*hooked*	0.5
	*T15*75-SFRC -2*	200	500	460	3248.8	0.0280	3.40	3.35	10.0	37.7	*hooked*	0.5
	*T15*50-SFRC -1*	200	500	460	3248.8	0.0280	3.40	3.35	10.0	37.7	*hooked*	0.5
	1.2/2	200	300	260	1948.32	0.0356	3.50	3.42	10.0	46.9	*hooked*	0.25
	1.2/3	200	300	260	1948.32	0.0356	3.50	3.42	10.0	43.7	*hooked*	0.51
	1.2/4	200	300	260	1948.32	0.0356	3.50	3.42	10.0	48.3	*hooked*	0.76
	*20*30-SFRC - 1*	200	300	260	1968.64	0.0283	3.50	3.46	10.0	37.7	*hooked*	0.5
	*20*30-SFRC - 2*	200	300	260	1968.64	0.0283	3.50	3.46	10.0	38.8	*hooked*	0.5
	*20*60-SFRC - 1*	200	600	540	3929.52	0.0273	3.50	3.48	10.0	37.7	*hooked*	0.25
	*20*60-SFRC - 2*	200	600	560	3929.52	0.0273	3.50	3.36	10.0	38.8	*hooked*	0.5
	2.6/2	200	300	260	2253.12	0.0181	4.00	4.01	10.0	41.2	*hooked*	0.25
	2.6/3	200	300	260	2253.12	0.0181	4.00	4.01	10.0	40.3	*hooked*	0.76
Sahoo et al. 2016	*TB0.75_1.6*	150	250	217	1150	0.0185	1.59	1.13	10.0	35	*hooked*	0.75
	TB0.75_2.5	150	250	217	1600	0.0185	2.47	2.00	10.0	35	*hooked*	0.75
	TB0.75_3.0	150	250	217	1750	0.0185	2.95	2.49	10.0	35	*hooked*	0.75

	B25-0-0-	300	700	622	4500	0.0198	2.81	2.49	10.0	34	*hooked*	0.321
	B50-0-0	300	700	622	4500	0.0198	2.81	2.49	10	36	*hooked*	0.687
	S0F0.5-65	100	150	135	900	0.0116	2.22	2.15	15.0	64.2	*hooked*	0.5
	S0F0.5-65	100	150	135	900	0.0116	2.22	2.15	15.0	64.2	*hooked*	0.5
Amin & Foster 2016 Tahenni et al. 2016	*S0F0.5-65*	100	150	135	900	0.0116	2.22	2.15	15.0	64.2	*hooked*	0.5
	S0F1.0-65	100	150	135	900	0.0116	2.22	2.15	15.0	64	*hooked*	1
	S0F1.0-65	100	150	135	900	0.0116	2.22	2.15	15.0	64	*hooked*	1
	S0F1.0-65	100	150	135	900	0.0116	2.22	2.15	15.0	64	*hooked*	1
	S0F1.0-80	100	150	135	900	0.0116	2.22	2.15	15.0	60	*hooked*	1
	S0F1.0-80	100	150	135	900	0.0116	2.22	2.15	15.0	60	*hooked*	1
	S0F1.0-80	100	150	135	900	0.0116	2.22	2.15	15.0	60	*hooked*	1
	SF1	85	150	130	900	0.0205	2.02	1.94	9.6	51.85	*crimped*	0.25
	SF2	85	150	130	1030	0.0205	2.52	2.44	9.6	51.85	*crimped*	0.25
	SF3	85	150	130	1160	0.0205	3.02	2.94	9.6	51.85	*crimped*	0.25
	SF4	85	150	130	900	0.0205	2.02	1.94	9.6	33.32	*crimped*	0.25
	SF5	85	150	130	1030	0.0205	2.52	2.44	9.6	33.32	*crimped*	0.25
	SF6	85	150	130	1160	0.0205	3.02	2.94	9.6	33.32	*crimped*	0.25
	B1	85	150	130	1160	0.0205	3.02	2.94	9.6	51.68	*crimped*	0.5
	B7	85	150	130	1160	0.0205	3.02	2.94	9.6	30.6	*crimped*	0.5
	B9	85	150	130	1160	0.0205	3.02	2.94	9.6	31.025	*crimped*	1
	B11	85	150	130	900	0.0205	2.02	1.94	9.6	51.68	*crimped*	0.5
	B12	85	150	130	1030	0.0205	2.52	2.44	9.6	51.68	*crimped*	0.5
	B13	85	150	130	1290	0.0205	3.52	3.44	9.6	41.65	*crimped*	0.5
	B14	85	150	130	900	0.0205	2.02	1.94	9.6	48.705	*crimped*	1
Narayanan & Darwish 1987	*B15*	85	150	130	1030	0.0205	2.52	2.44	9.6	48.705	*crimped*	1
	B16	85	150	130	1290	0.0205	3.52	3.44	9.6	48.79	*crimped*	1
	B17	85	150	128	1160	0.0370	3.06	2.98	9.6	41.65	*crimped*	0.5
	B18	85	150	126	1160	0.0572	3.11	3.03	9.6	41.65	*crimped*	0.5
	B19	85	150	128	1160	0.0370	3.06	2.98	9.6	30.6	*crimped*	0.5
	B20	85	150	126	1160	0.0572	3.11	3.03	9.6	30.6	*crimped*	0.5
	B23	85	150	128	1160	0.0370	3.06	2.98	9.6	48.79	*crimped*	1
	B24	85	150	126	1160	0.0572	3.11	3.03	9.6	48.79	*crimped*	1
	B25	85	150	126	1160	0.0572	3.11	3.03	9.6	53.55	*crimped*	1.5
	B26	85	150	126	1160	0.0572	3.11	3.03	9.6	43.18	*crimped*	2
	B27	85	150	128	1160	0.0370	3.06	2.98	9.6	53.55	*crimped*	1.5
	B28	85	150	126	900	0.0572	2.08	2.00	9.6	50.15	*crimped*	0.5
	B29	85	150	126	900	0.0572	2.08	2.00	9.6	45.9	*crimped*	1
	B30	85	150	126	900	0.0572	2.08	2.00	9.6	53.55	*crimped*	1.5
	B31	85	150	126	900	0.0572	2.08	2.00	9.6	43.18	*crimped*	2
	A10	150	250	219	2300	0.0191	2.80	2.75	10.0	40.85	*hooked*	1
Cucchiara et al. 2004	*A20*	150	250	219	2300	0.0191	2.80	2.75	10.0	40.85	*hooked*	2
	B10	150	250	219	2300	0.0191	2.00	1.95	10.0	43.23	*hooked*	1
	B20	150	250	219	2300	0.0191	2.00	1.95	10.0	43.23	*hooked*	2

**Kwak et al. 2002**	*FHB2-2*	125	250	212	1248	0.0152	2.00	1.46	19.0	63.8	*hooked*	0.5
	FHB3-2	125	250	212	1248	0.0152	2.00	1.46	19.0	68.6	*hooked*	0.75
	FNB2-2	125	250	212	1248	0.0152	2.00	1.46	19.0	30.8	*hooked*	0.5
	FNB2-3	125	250	212	1672	0.0152	3.00	2.46	19.0	30.8	*hooked*	0.5
**Lim & Oh 1999**	*S0.00V1*	100	180	130	1300	0.0309	3.08	3.00	10.0	38.69	*straight smooth*	1
	S0.00V2	100	180	130	1300	0.0309	3.08	3.00	10.0	42.4	*straight smooth*	2
**Dinh et al. 2010**	*B18-1a*	152	455	381	2136	0.0196	3.44	3.04	10.0	44.8	*hooked*	0.75
	B18-1b	152	455	381	2136	0.0196	3.44	3.04	10.0	44.8	*hooked*	0.75
	B18-2a	152	455	381	2136	0.0196	3.44	3.04	10.0	38.1	*hooked*	1
	B18-2b	152	455	381	2136	0.0196	3.44	3.04	10.0	38.1	*hooked*	1
	B18-3a	152	455	381	2136	0.0263	3.44	3.04	10.0	31	*hooked*	1.5
	B18-3b	152	455	381	2136	0.0263	3.44	3.04	10.0	31	*hooked*	1.5
	B18-3c	152	455	381	2136	0.0263	3.44	3.04	10.0	44.9	*hooked*	1.5
	B18-3d	152	455	381	2136	0.0263	3.44	3.04	10.0	44.9	*hooked*	1.5
	B18-5a	152	455	381	2136	0.0263	3.44	3.04	10.0	49.2	*hooked*	1
	B18-5b	152	455	381	2136	0.0263	3.44	3.04	10.0	49.2	*hooked*	1
	B18-7a	152	455	381	2136	0.0196	3.44	3.04	10.0	43.3	*hooked*	0.75
	B18-7b	152	455	381	2136	0.0196	3.44	3.04	10.0	43.3	*hooked*	0.75
	B27-1a	205	685	610	3558	0.0196	3.50	3.21	10.0	50.8	*hooked*	0.75
	B27-1b	205	685	610	3558	0.0196	3.50	3.21	10.0	50.8	*hooked*	0.75
	B27-2a	205	685	610	3558	0.0196	3.50	3.21	10.0	28.7	*hooked*	0.75
	B27-2b	205	685	610	3558	0.0196	3.50	3.21	10.0	28.7	*hooked*	0.75
	B27-3b	205	685	610	3558	0.0152	3.50	3.21	10.0	42.3	*hooked*	0.75
	B27-4a	205	685	610	3558	0.0152	3.50	3.21	10.0	29.6	*hooked*	0.75
	B27-4b	205	685	610	3558	0.0152	3.50	3.21	10.0	29.6	*hooked*	0.75
	B27-5	205	685	610	3558	0.0196	3.50	3.21	10.0	44.4	*hooked*	1.5
	B27-6	205	685	610	3558	0.0196	3.50	3.21	10.0	42.8	*hooked*	1.5
**Lima Araujo et al. 2014**	*V-1-0*	150	390	340	2200	0.0308	2.50	2.21	12.5	58.87	*hooked*	1
	V-2-0	150	390	340	2200	0.0308	2.50	2.21	12.5	51.67	*hooked*	2
**Casanova et al. 1997**	*FRC1*	150	800	735	5600	0.0106	3.81	3.67	12.5	42	*hooked*	1.25
	FRC2	150	800	735	5600	0.0106	3.81	3.67	12.5	38	*hooked*	1.25
	HSFRC 1	125	250	225	2000	0.0349	2.89	2.44	10.0	90	*hooked*	1.25
**Aoude et al. 2012**	*A0.5%*	150	250	202	1700	0.0117	2.97	2.48	10.0	21.3	*hooked*	0.5
	A1%	150	250	202	1700	0.0117	2.97	2.48	10.0	19.6	*hooked*	1
	B0.5%	300	500	437	3700	0.015	3.09	2.86	10.0	21.3	*hooked*	0.5
	B1%	300	500	437	3700	0.015	3.09	2.86	10	19.6	*hooked*	1
**Minelli & Plizzari 2013**	*NSC1-FRC1*	200	480	435	4350	0.0104	2.51	2.30	20	24.8	*hooked*	0.38
	NSC2-FRC1	200	480	435	4350	0.0104	2.51	2.30	20	33.5	*hooked*	0.38
	NSC2-FRC2	200	480	435	4350	0.0104	2.51	2.30	20	33.5	*hooked + straight*	0.57
	NSC3-FRC	200	480	435	4350	0.0104	2.51	2.30	20	38.6	*hooked*	0.38
	HSC1-FRC1	200	480	435	4350	0.0104	2.51	2.30	20	61.1	*hooked*	0.64
	NSC4-FRC-500-1	200	500	455	2280	0.0099	2.51	2.31	15	24.4	*hooked*	0.25
	NSC4-FRC-500-2	200	500	455	2280	0.0099	2.51	2.31	15	24.4	*hooked*	0.25
	NSC4-FRC-1000	200	1000	910	4550	0.0104	2.50	2.40	20	24.4	*hooked*	0.25
	HSC2-FRC-1000	200	1000	910	4550	0.0104	2.50	2.40	20	55	*hooked*	0.25

Kang et al. 2011	*FLB-0.5-2*	125	250	210	1250	0.0153	2.00	1.52	19	44.6	*hooked*	0.5
	FLB-0.5-4	125	250	210	2100	0.0153	4.00	3.52	19	44.6	*hooked*	0.5
	FNB-0.5-2	125	250	210	1250	0.0153	2.00	1.52	19	57.2	*hooked*	0.5
Casanova & Rossi 1999	*HSFRC 1*	125	250	225	2000	0.0349	2.89	2.44	10	90	*hooked*	1.25
	HSFRC 2	125	250	225	2000	0.0349	2.89	2.44	10	90	*hooked*	1.25
Lim et al. 1987	*2/0.5/2.5*	152	254	221	2100	0.0120	2.50	2.45	10	34	*hooked*	0.5
	4/1.0/1.5	152	254	221	1600	0.0239	1.50	1.45	10	34	*hooked*	1
	4/1.0/2.5	152	254	221	2100	0.0239	2.50	2.45	10	34	*hooked*	1
	4/1.0/3.5	152	254	221	2100	0.0239	3.50	3.45	10	34	*hooked*	1
	4/0.5/1.5	152	254	221	1600	0.0239	1.50	1.45	10	34	*hooked*	0.5
	4/0.5/2.5	152	254	221	2100	0.0239	2.50	2.45	10	34	*hooked*	0.5
	4/0.5/3.5	152	254	221	2100	0.0239	3.50	3.45	10	34	*hooked*	0.5
Mansur et al. 1986	*B1*	150	225	197	1288	0.0136	2.00	1.49	20	29.1	*hooked*	0.5
	B2	150	225	197	1603.2	0.0136	2.80	2.29	20	29.1	*hooked*	0.5
	B3	150	225	197	1918.4	0.0136	3.60	3.09	20	29.1	*hooked*	0.5
	C1	150	225	197	1288	0.0136	2.00	1.49	20	29.9	*hooked*	0.75
	C2	150	225	197	1603.2	0.0136	2.80	2.29	20	29.9	*hooked*	0.75
	C6	150	225	197	1603.2	0.0204	2.80	2.29	20	29.9	*hooked*	0.75
	E2	150	225	197	1603.2	0.0136	2.80	2.29	20	20.6	*hooked*	0.75
	E3	150	225	197	1603.2	0.0204	2.80	2.29	20	20.6	*hooked*	0.75
	F3	150	225	197	1603.2	0.0204	2.80	2.29	20	33.4	*hooked*	0.75
Zarrinpour & Chao 2017	*SFRC 12W6*	152	305	254	1778	0.0248	3.50	2.90	10	29	*hooked*	0.75
	SFRC 12W24	610	305	254	1778	0.0247	3.50	2.90	10	29	*hooked*	0.75
	SFRC 18a	152	457	394	2844.8	0.0286	3.61	3.22	10	39	*hooked*	0.75
	SFRC 18b	152	457	394	2844.8	0.0286	3.61	3.22	10	39	*hooked*	0.75
	SFRC 24a	203	610	541	3733.8	0.0254	3.45	3.17	10	50	*hooked*	0.75
	SFRC 24b	203	610	541	3733.8	0.0254	3.45	3.17	10	50	*hooked*	0.75
	SFRC 36a	254	915	813	5689.6	0.0270	3.50	3.31	10	50	*hooked*	0.75
	SFRC 36b	254	915	813	5689.6	0.0270	3.50	3.31	10	50	*hooked*	0.75
	SFRC 48a	305	1220	1118	7823.2	0.0255	3.50	3.35	10	50	*hooked*	0.75
	SFRC 48b	305	1220	1118	7823.2	0.0255	3.50	3.35	10	50	*hooked*	0.75
Noghabai 2000	*HSC.I.S6.0.15*	200	250	180	1200	0.0447	3.33	2.22	16	90.6	*straight smooth*	1
	HSC.I.Smix	200	250	180	1200	0.0447	3.33	2.22	16	83.2	*hooked + straight*	1
	HSC.I.S60/0.7/0.5	200	250	180	1200	0.0447	3.33	2.22	16	80.5	*hooked*	0.5
	HSC.I.S60/0.7/0.75	200	250	180	1200	0.0447	3.33	2.22	16	80.5	*hooked*	0.75
	NSC.II.Smix	200	250	195	1200	0.0309	3.08	2.05	16	39.4	*hooked + straight*	1
	HSC.II.S30/0.6	200	300	235	1300	0.0428	2.77	1.91	16	91.4	*hooked*	1
	HSC.II.S6/0.15	200	300	235	1300	0.0428	2.77	1.91	16	93.3	*straight smooth*	1
	HSC.II.Smix	200	300	235	1300	0.0428	2.77	1.91	16	89.6	*hooked + straight*	1
	HSC.III.S6/0.15	200	500	410	3000	0.0306	2.93	2.44	18	76.8	*straight smooth*	1
	HSC.III.S6/0.15	200	500	410	3000	0.0306	2.93	2.44	18	76.8	*straight smooth*	1
	HSC.III.Smix	200	500	410	3000	0.0306	2.93	2.44	18	72	*hooked + straight*	1
	HSC.III.Smix	200	500	410	3000	0.0306	2.93	2.44	18	72	*hooked + straight*	1
	HSC.III.S60/0.7/0.5	200	500	410	3000	0.0306	2.93	2.44	18	69.3	*hooked*	0.5
	HSC.III.S60/0.7/0.5	200	500	410	3000	0.0306	2.93	2.44	18	69.3	*hooked*	0.5

	HSC.III.S60/0.7/0.75	200	500	410	3000	0.0306	2.93	2.44	18	60.2	*hooked*	0.75
	HSC.IV.S60/0.7/0.75	200	500	410	3000	0.0306	2.93	2.44	18	75.7	*hooked*	0.75
	HSC.III.S6/0.15	300	700	570	5000	0.0287	2.98	2.63	18	76.8	*straight smooth*	1
	HSC.IV.Smix	300	700	570	5000	0.0287	2.98	2.63	18	72	*hooked + straight*	1
	HSC.III.S60/0.7/0.75	300	700	570	5000	0.0287	2.98	2.63	18	60.2	*hooked*	0.75
Randl et al. 2017	*B19*	200	350	314	3000	0.0350	3.50	3.18	0.4	132	*straight smooth*	2
	B25	200	350	314	3000	0.0350	3.50	3.18	0.4	154	*straight smooth*	2
	B30	200	350	314	3000	0.0350	3.50	3.18	0.4	146	*straight smooth*	2
	B20	200	350	314	3000	0.0350	3.50	3.18	0.4	133	*straight smooth*	1
	B24	200	350	314	3000	0.0350	3.50	3.18	0.4	143	*straight smooth*	1
	B29	200	350	314	3000	0.0350	3.50	3.18	0.4	153	*straight smooth*	1
Ashour et al. 1992	*B-2-1.0-L*	125	250	215	1360	0.0037	2.00	1.95	10	92	*hooked*	1
	B-4-1.0-L	125	250	215	2220	0.0037	4.00	3.95	10	92.6	*hooked*	1
	B-6-1.0-L	125	250	215	3080	0.0037	6.00	5.95	10	93.7	*hooked*	1
	B-1-0.5-A	125	250	215	930	0.0283	1.00	0.95	10	99	*hooked*	0.5
	B-2-0.5-A	125	250	215	1360	0.0283	2.00	1.95	10	99.1	*hooked*	0.5
	B-4-0.5-A	125	250	215	2220	0.0283	4.00	3.95	10	95.4	*hooked*	0.5
	B-6-0.5-A	125	250	215	3080	0.0283	6.00	5.95	10	95.83	*hooked*	0.5
	B-1-1.0-A	125	250	215	930	0.0283	1.00	0.95	10	95.3	*hooked*	1
	B-2-1.0-A	125	250	215	1360	0.0283	2.00	1.95	10	95.3	*hooked*	1
	B-4-1.0-A	125	250	215	2220	0.0283	4.00	3.95	10	97.53	*hooked*	1
	B-6-1.0-A	125	250	215	3080	0.0283	6.00	5.95	10	100.5	*hooked*	1
	B-1-1.5-A	125	250	215	930	0.0283	1.00	0.95	10	96.4	*hooked*	1.5
	B-2-1.5-A	125	250	215	1360	0.0283	2.00	1.95	10	96.6	*hooked*	1.5
	B-4-1.5-A	125	250	215	2220	0.0283	4.00	3.95	10	97.1	*hooked*	1.5
	B-6-1.5-A	125	250	215	3080	0.0283	6.00	5.95	10	101.32	*hooked*	1.5
	B-2-1.0-M	125	250	215	1360	0.0458	2.00	1.95	10	94.5	*hooked*	1
	B-4-1.0-M	125	250	215	2220	0.0458	4.00	3.95	10	93.8	*hooked*	1
	B-6-1.0-M	125	250	215	3080	0.0458	6.00	5.95	10	95	*hooked*	1
Tan et al. 1993	*2*	140	375	340	1910	0.0167	2.00	1.71	19	35	*hooked*	0.5
	3	140	375	340	1910	0.0167	2.00	1.71	19	33	*hooked*	0.75
	4	140	375	340	1910	0.0167	2.00	1.71	19	36	*hooked*	1
	5	140	375	340	1910	0.0167	2.50	2.21	19	36	*hooked*	1
	6	140	375	340	1910	0.0167	1.50	1.21	19	36	*hooked*	1
Pansuk et al. 2017	*NS08*	150	400	350	2000	0.0561	2.86	2.57	2	121.1058	*hooked*	0.8
	NS16	150	400	350	2000	0.0561	2.86	2.57	2	120.3022	*hooked*	1.6
Kim et al. 2017	*21FB*	260	400	340	3120	0.0172	4.00	3.71	10	21	*hooked*	0.75
	60FB	260	400	340	3120	0.0172	4.00	3.71	10	56	*hooked*	0.75
Sharma, 1986	*S3F*	150	300	276	1600	0.0146	1.81	1.78	10	48.6	*hooked*	0.96
Narayanan & Darwish 1988	*D2*	100	400	345	1000	0.0355	0.70	0.43	5	52.89	*crimped*	0.25
	D3	100	400	345	1000	0.0355	0.70	0.43	5	51.004	*crimped*	0.5
	D4	100	400	345	1000	0.0355	0.70	0.43	5	47.56	*crimped*	0.75
	D5	100	400	345	1000	0.0355	0.70	0.43	5	55.924	*crimped*	1
	D6	100	400	345	1000	0.0355	0.70	0.43	5	54.94	*crimped*	1.25
	D7	100	400	345	1000	0.0355	0.46	0.20	5	50.512	*crimped*	1

	D8	100	400	345	1000	0.0355	0.58	0.32	5	47.806	*crimped*	1
	D9	100	400	345	1000	0.0355	0.81	0.55	5	45.592	*crimped*	1
	D10	100	400	345	1000	0.0355	0.93	0.67	5	49.118	*crimped*	1
	D11	100	400	345	1000	0.0355	0.70	0.43	5	30.996	*crimped*	1
	D12	100	400	345	1000	0.0355	0.70	0.43	5	34.686	*crimped*	1
Li, Ward & Hamza 1992	*M1*	63.5	127	102	612	0.0220	3.00	2.88	2.36	53	*crimped*	1
	M2	127	228	204	1224	0.0221	3.00	2.88	2.36	53	*crimped*	1
	M3	63.5	127	102	612	0.0220	3.00	2.88	2.36	50.2	*crimped*	2
	M4	127	228	204	1224	0.0221	3.00	2.88	2.36	50.2	*crimped*	2
	M5	63.5	127	102	612	0.0220	3.00	2.88	2.36	62.6	*crimped*	1
	M6	127	228	204	1224	0.0221	3.00	2.88	2.36	62.6	*crimped*	1
	M8	63.5	127	102	204	0.0220	1.00	0.88	2.36	62.6	*crimped*	1
	M9	63.5	127	102	306	0.0220	1.50	1.38	2.36	62.6	*crimped*	1
	M10	63.5	127	102	357	0.0220	1.75	1.63	2.36	62.6	*crimped*	1
	M11	63.5	127	102	408	0.0220	2.00	1.88	2.36	62.6	*crimped*	1
	M12	63.5	127	102	459	0.0220	2.25	2.13	2.36	62.6	*crimped*	1
	M13	63.5	127	102	510	0.0220	2.50	2.38	2.36	62.6	*crimped*	1
	M14	63.5	127	102	561	0.0220	2.75	2.63	2.36	62.6	*crimped*	1
	M15	63.5	127	102	612	0.0110	3.00	2.88	2.36	62.6	*crimped*	1
	M16	63.5	127	102	612	0.0330	3.00	2.88	2.36	62.6	*crimped*	1
	M17	63.5	127	102	612	0.0330	3.00	2.88	2.36	54.1	*crimped*	1
	C1	127	228	204	1224	0.0221	3.00	2.88	9	22.7	*hooked*	1
	C2	63.5	127	102	612	0.0220	3.00	2.88	9	22.7	*hooked*	1
	C3	63.5	127	102	612	0.0110	3.00	2.88	9	22.7	*hooked*	1
	C4	63.5	127	102	306	0.0110	1.50	1.38	9	22.7	*hooked*	1
	C5	127	228	204	1224	0.0221	3.00	2.88	9	26	*hooked*	1
	C6	63.5	127	102	612	0.0220	3.00	2.88	9	26	*hooked*	1
Swamy et al. 1993	*1TLF-1*	55	300	265	3000	0.0431	2.00	1.62	14	36.49	*crimped*	1
	1TLF-2	55	300	265	3000	0.0431	3.43	3.05	14	41.902	*crimped*	1
	1TLF-3	55	300	265	3000	0.0431	4.91	4.53	14	36.9	*crimped*	1
	2TLF-1	55	300	265	3000	0.0276	2.00	1.62	14	38.704	*crimped*	1
	2TLF-2	55	300	265	3000	0.0276	3.43	3.05	14	33.948	*crimped*	1
	2TLF-3	55	300	265	3000	0.0276	4.91	4.53	14	36.818	*crimped*	1
	3TLF-1	55	300	265	3000	0.0155	2.00	1.62	14	36.572	*crimped*	1
Adebar et al. 1997	*FC2*	150	610	560	1500	0.0214	1.63	1.34	14	54.1	*hooked*	0.75
	FC3	150	610	560	1500	0.0214	1.63	1.34	14	49.9	*hooked*	1.5
	FC8	150	610	560	1500	0.0214	1.63	1.34	14	54.8	*hooked*	0.4
	FC9	150	610	560	1500	0.0214	1.63	1.34	14	56.5	*hooked*	0.6
	FC10	150	610	560	1500	0.0214	1.63	1.34	14	46.9	*hooked*	0.4
	FC11	150	610	560	1500	0.0214	1.63	1.34	14	40.8	*hooked*	0.6

Cho & Kim 2003	*F30-0.5-13*	120	200	167.5	720	0.0132	1.43	1.25	13	25.7	*hooked*	0.5
	F30-1.0-13	120	200	167.5	720	0.0132	1.43	1.25	13	25.3	*hooked*	1
	F30-1.5-13	120	200	167.5	720	0.0132	1.43	1.25	13	23.9	*hooked*	1.5
	F50-0.5-13	120	200	167.5	720	0.0132	1.43	1.25	13	57.8	*hooked*	0.5
	F60-1.0-13	120	200	167.5	720	0.0132	1.43	1.25	13	61.5	*hooked*	1
	F70-0.5-19	120	200	167.5	720	0.0282	1.43	1.25	13	70.5	*hooked*	0.5
	F70-1.0-19	120	200	167.5	720	0.0282	1.43	1.25	13	67.3	*hooked*	1
	F70-1.5-19	120	200	167.5	720	0.0282	1.43	1.25	13	67.3	*hooked*	1.5
	F80-0.5-16	120	200	167.5	720	0.0200	1.43	1.25	13	82.4	*hooked*	0.5
	F80-1.0-16	120	200	167.5	720	0.0200	1.43	1.25	13	81.1	*hooked*	1
	F80-0.5-19	120	200	167.5	720	0.0282	1.43	1.25	13	86.1	*hooked*	0.5
	F80-1.0-19	120	200	167.5	720	0.0282	1.43	1.25	13	89.4	*hooked*	1
Greenough & Nehdi 2008	*S-HE-50-0.5*	200	300	265	2000	0.0178	3.02	2.64	10	47.9	*hooked*	0.5
	S-HE-50-0.75	200	300	265	2000	0.0178	3.02	2.64	10	38	*hooked*	0.75
	S-HE-50-1.0	200	300	265	2000	0.0178	3.02	2.64	10	42.2	*hooked*	1
	S-FE-50-0.5	200	300	265	2000	0.0178	3.02	2.64	10	45.4	*flat end*	0.5
	S-FE-50-0.75	200	300	265	2000	0.0178	3.02	2.64	10	44.4	*flat end*	0.75
	S-FE-50-1.0	200	300	265	2000	0.0178	3.02	2.64	10	40.3	*flat end*	1
	S-FE-30-0.5	200	300	265	2000	0.0178	3.02	2.64	10	53.7	*flat end*	0.5
	S-FE-30-0.75	200	300	265	2000	0.0178	3.02	2.64	10	46	*flat end*	0.75
	S-FE-30-1.0	200	300	265	2000	0.0178	3.02	2.64	10	42.2	*flat end*	1
Kang et al. 2012	*FNB-50-1*	200	355	310	3560	0.0113	2.55	1.98	9.5	39.8	*hooked*	0.375
	FNB-50-3	200	355	285	3560	0.0333	2.77	2.16	9.5	39.8	*hooked*	0.375
Dupont & Vandewalle 2003	*2*	200	300	260	1800	0.0355	3.46	3.08	14	46.4	*hooked*	0.25
	3	200	300	260	1800	0.0355	3.46	3.08	14	43.2	*hooked*	0.5
	4	200	300	260	1800	0.0355	3.46	3.08	14	47.6	*hooked*	0.75
	14	200	300	260	2300	0.0181	1.54	1.15	14	40.7	*hooked*	0.25
	15	200	300	260	2300	0.0181	1.54	1.15	14	42.4	*hooked*	0.75
	17	200	300	262	2300	0.0115	2.48	2.10	14	39.1	*hooked*	0.25
	18	200	300	262	2300	0.0115	2.48	2.10	14	38.6	*hooked*	0.75
	20	200	300	260	2300	0.0181	2.50	2.12	14	39.1	*hooked*	0.25
	21	200	300	260	2300	0.0181	2.50	2.12	14	38.6	*hooked*	0.75
	23	200	300	260	2300	0.0181	4.04	3.65	14	40.7	*hooked*	0.25
	24	200	300	260	2300	0.0181	4.04	3.65	14	42.4	*hooked*	0.75
	26	200	300	262	2300	0.0115	2.48	2.10	14	26.5	*hooked*	0.25
	27	200	300	262	2300	0.0115	2.48	2.10	14	27.2	*hooked*	0.75
	29	200	300	260	2300	0.0181	2.50	2.12	14	26.5	*hooked*	0.25
	30	200	300	260	2300	0.0181	2.50	2.12	14	27.2	*hooked*	0.75
	31	200	300	262	2300	0.0115	2.48	2.10	14	47.4	*hooked*	0.5
	32	200	300	260	2300	0.0181	2.50	2.12	14	46.8	*hooked*	0.5
	33	200	300	262	2300	0.0115	2.48	2.10	14	45.4	*hooked*	0.5
	41	200	350	305	3250	0.0103	2.46	2.13	14	34.4	*hooked*	0.57
	43	200	350	305	3250	0.0103	2.46	2.13	14	30.2	*hooked*	0.38

**Swamy & Bahia 1985**	*B52*	175	250	210	2800	0.0401	4.50	4.26	10	36.408	*crimped*	0.4
	B53	175	250	210	2800	0.0401	4.50	4.26	10	38.376	*crimped*	0.8
	B54	175	250	210	2800	0.0401	4.50	4.26	10	40.836	*crimped*	1.2
	B55	175	250	210	2800	0.0310	4.50	4.26	10	39.114	*crimped*	0.8
	B59	175	250	210	2800	0.0401	4.50	4.26	10	38.54	*crimped*	0.8
**Batson et al. 1972**	*H1*	101	152	127	1828.8	0.0309	4.80	4.01	2	33.22	*flat*	0.22
	H2	101	152	127	1828.8	0.0309	4.80	4.01	2	33.22	*flat*	0.22
	H3	101	152	127	1828.8	0.0309	4.80	4.01	2	33.22	*flat*	0.22
	I1	101	152	127	1828.8	0.0309	4.80	4.01	2	33.22	*crimped*	0.22
	I2	101	152	127	1828.8	0.0309	4.80	4.01	2	33.22	*crimped*	0.22
	I3	101	152	127	1828.8	0.0309	4.80	4.01	2	33.22	*crimped*	0.22
	A2	101	152	127	1828.8	0.0309	4.80	4.01	2	33.22	*round*	0.22
	B3	101	152	127	1828.8	0.0309	4.40	3.61	2	33.22	*round*	0.22
	C1	101	152	127	1828.8	0.0309	4.20	3.41	2	33.22	*round*	0.22
	C2	101	152	127	1828.8	0.0309	4.20	3.41	2	33.22	*round*	0.22
	C3	101	152	127	1828.8	0.0309	4.20	3.41	2	33.22	*round*	0.22
	D2	101	152	127	1828.8	0.0309	4.30	3.51	2	33.22	*round*	0.22
	D3	101	152	127	1828.8	0.0309	4.30	3.51	2	33.22	*round*	0.22
	E3	101	152	127	1828.8	0.0309	4.20	3.41	2	40.21	*round*	0.44
	F1	101	152	127	1828.8	0.0309	4.00	3.21	2	40.21	*round*	0.44
	F2	101	152	127	1828.8	0.0309	4.00	3.21	2	40.21	*round*	0.44
	F3	101	152	127	1828.8	0.0309	4.00	3.21	2	40.21	*round*	0.44
	G1	101	152	127	1828.8	0.0309	4.40	3.61	2	33.22	*round*	0.22
	G3	101	152	127	1828.8	0.0309	4.40	3.61	2	33.22	*round*	0.22
	L1	101	152	127	1828.8	0.0309	4.00	3.21	2	33.22	*crimped*	0.22
	L2	101	152	127	1828.8	0.0309	4.00	3.21	2	33.22	*crimped*	0.22
	L3	101	152	127	1828.8	0.0309	4.00	3.21	2	33.22	*crimped*	0.22
	M1	101	152	127	1828.8	0.0309	4.60	3.81	2	33.22	*crimped*	0.22
	M2	101	152	127	1828.8	0.0309	4.40	3.61	2	33.22	*crimped*	0.22
	M3	101	152	127	1828.8	0.0309	4.40	3.61	2	33.22	*crimped*	0.22
	N1	101	152	127	1828.8	0.0309	5.00	4.21	2	33.22	*crimped*	0.22
	N2	101	152	127	1828.8	0.0309	4.80	4.01	2	33.22	*crimped*	0.22
	O1	101	152	127	1828.8	0.0309	4.00	3.21	2	40.21	*crimped*	0.44
	P1	101	152	127	1828.8	0.0309	4.20	3.41	2	40.21	*crimped*	0.44
	P2	101	152	127	1828.8	0.0309	4.20	3.41	2	40.21	*crimped*	0.44
	P3	101	152	127	1828.8	0.0309	4.20	3.41	2	40.21	*crimped*	0.44
	R1	101	152	127	1828.8	0.0309	3.20	2.41	2	39.72	*crimped*	0.88
	R2	101	152	127	1828.8	0.0309	3.40	2.61	2	39.72	*crimped*	0.88
	S1	101	152	127	1828.8	0.0309	3.40	2.61	2	39.72	*crimped*	0.88
	S2	101	152	127	1828.8	0.0309	3.40	2.61	2	39.72	*crimped*	0.88
	S3	101	152	127	1828.8	0.0309	3.40	2.61	2	39.72	*crimped*	0.88
	U1	101	152	127	1828.8	0.0309	2.80	2.01	2	39.79	*crimped*	1.76
	V2	101	152	127	1828.8	0.0309	1.80	1.01	2	39.79	*crimped*	1.76
	W1	101	152	127	1828.8	0.0309	1.20	0.41	2	39.79	*crimped*	1.76
	W2	101	152	127	1828.8	0.0309	1.20	0.41	2	39.79	*crimped*	1.76
	X1	101	152	127	1828.8	0.0309	4.80	4.01	2	33.22	*crimped*	0.22

	X2	101	152	127	1828.8	0.0309	4.80	4.01	2	33.22	*crimped*	0.22
	X3	101	152	127	1828.8	0.0309	4.80	4.01	2	33.22	*crimped*	0.22
Zhao et al. 2018	*S0005*	150	300	259.5	2100	0.0252	2.00	1.61	20	34.45	*mill-cut*	0.5
	S0010	150	300	259.5	2100	0.0252	2.00	1.61	20	36.08	*mill-cut*	1
	S0015	150	300	259.5	2100	0.0252	2.00	1.61	20	37.13	*mill-cut*	1.5
	S0020	150	300	259.5	2100	0.0252	2.00	1.61	20	35.26	*mill-cut*	2
Jindal 1984	*C1*	100	152.6	127	1524	0.0199	3.60	3.52	2	20.68966	*straight mild steel*	1
	G1	100	152.6	127	762	0.0199	2.00	1.92	2	20.68966	*brass-coated high strength steel*	1
	G2	100	152.6	127	762	0.0199	2.40	2.32	2	20.68966	*brass-coated high strength steel*	1
	H1	100	152.6	127	762	0.0199	2.00	1.92	2	20.68966	*brass-coated high strength steel*	1
	H3	100	152.6	127	1524	0.0199	3.60	3.52	2	20.68966	*brass-coated high strength steel*	1
	H4	100	152.6	127	1524	0.0199	4.80	4.72	2	20.68966	*brass-coated high strength steel*	1
	J1	100	152.6	127	762	0.0199	2.00	1.92	2	20.68966	*brass-coated high strength steel*	1
Shin, Oh & Ghosh 1994	*2-0.5-0.5*	100	200	175	700	0.0359	2.00	1.94	13	80	*smooth straight*	0.5
	2-0.5-1	100	200	175	700	0.0359	2.00	1.94	13	80	*smooth straight*	1
	3-0.5-0.5	100	200	175	1050	0.0359	3.00	2.94	13	80	*smooth straight*	0.5
	3-0.5-1	100	200	175	1050	0.0359	3.00	2.94	13	80	*smooth straight*	1
	4.5-0.5-0.5	100	200	175	1575	0.0359	4.50	4.44	13	80	*smooth straight*	0.5
	4.5-0.5-1	100	200	175	1575	0.0359	4.50	4.44	13	80	*smooth straight*	1
Imam, Vandewalle & Mortelmans 1994	*B16*	200	350	300	3250	0.0308	1.75	1.42	10	109.5	*hooked*	0.75
	B6	200	350	300	3250	0.0308	2.50	2.17	10	110	*hooked*	0.75
	B7	200	350	300	3250	0.0308	3.50	3.17	10	111.5	*hooked*	0.75
	B12	200	350	300	3250	0.0308	4.50	4.17	10	110.8	*hooked*	0.75
Huang et al. 2005 [106]	*PB14*	150	300	255	2000	0.0493	1.96	1.57	20	55.842	*chopped with butt ends*	1
Kwak, Suh & Hsu 1991	*IAS1*	152.4	304.8	282.575	1524	0.0199	2.50	2.14	9.525	33.06897	*hooked*	1
	IAS2	152.4	304.8	282.575	1524	0.0199	2.50	2.14	9.525	33.24138	*hooked*	1
	IBS1	152.4	304.8	282.575	1524	0.0199	2.50	2.14	9.525	33.03448	*hooked*	2
	IBS2	152.4	304.8	282.575	1524	0.0199	2.50	2.14	9.525	34.37931	*hooked*	2
Roberts & Ho 1982	*F3.0B1*	50	200	170	820	0.0237	2.41	2.12	10	32.062	*brass-coated high strength steel*	3
	F4.5B1	50	200	170	820	0.0237	2.41	2.12	10	39.278	*brass-coated high strength steel*	4.5
	F3.0B2	50	200	170	552	0.0237	1.62	1.33	10	32.062	*brass-coated high strength steel*	3
	F4.5B2	50	200	170	552	0.0237	1.62	1.33	10	39.278	*brass-coated high strength steel*	4.5
	F3.0B3	50	200	170	274	0.0237	0.81	0.51	10	32.062	*brass-coated high strength steel*	3
	F4.5B3	50	200	170	274	0.0237	0.81	0.51	10	39.278	*brass-coated high strength steel*	4.5
Hwang et al. 2013	*S-35-0.5*	100	200	165.5	1500	0.0343	3.02	2.96	10	39.4	*hooked*	0.5
	S-35-1.0	100	200	165.5	1500	0.0343	3.02	2.96	10	39.2	*hooked*	1
	S-35-1.5	100	200	165.5	1500	0.0343	3.02	2.96	10	40	*hooked*	1.5
	S-35-2.0	100	200	165.5	1500	0.0343	3.02	2.96	10	35.5	*hooked*	2
	HS-50-1.0	100	200	159	1500	0.0478	3.14	3.08	10	58	*hooked*	1
	HS-70-00.5	100	200	159	1500	0.0478	3.14	3.08	10	80.1	*hooked*	0.5
	HS-70-1.0	100	200	159	1500	0.0478	3.14	3.08	10	88	*hooked*	1

Spinella et al.2012	*A10*	150	250	219	2300	0.0191	2.80	2.75	10	80.04	*hooked*	1
	B10	150	250	219	2300	0.0191	2.00	1.95	10	80.04	*hooked*	1
Chalioris & Sfiri, 2011	*MF40*	100	300	275	1450	0.0055	2.00	1.96	9.5	28.4	*hooked*	0.5
Cohen & Aoude, 2012	*M15-0.5%*	125	250	212	2400	0.0152	3.77	3.07	10	59.4	*hooked*	0.5
Aoude & Cohen 2014	*M15-0.5%H*	125	250	212	2400	0.0152	3.77	3.07	10	49.6	*hooked*	0.5
	M20-0.75%	125	250	210	2400	0.0228	3.81	3.10	10	49.7	*hooked*	0.75
	M20-1.0%	125	250	210	2400	0.0228	3.81	3.10	10	51.5	*hooked*	1
	M20-1.0%A	125	250	210	2400	0.0228	3.81	3.10	12	54.5	*hooked*	1
Qissab & Salman 2018	*G1B2*	100	170	140	1100	0.0112	1.07	0.50	12.5	36.08	*hooked*	0.5
	G1B3	100	170	140	1100	0.0112	1.07	0.50	12.5	36.9	*hooked*	0.75
	G1B5	100	170	140	1100	0.0112	2.50	1.93	12.5	36.08	*hooked*	0.5
	G1B6	100	170	140	1100	0.0112	2.50	1.93	12.5	36.9	*hooked*	0.75
	G2B2	100	180	150	1100	0.0105	1.00	0.47	12.5	36.08	*hooked*	0.5
	G2B3	100	180	150	1100	0.0105	1.00	0.47	12.5	36.9	*hooked*	0.75
	G2B5	100	180	150	1100	0.0105	2.33	1.80	12.5	36.08	*hooked*	0.5
	G2B6	100	180	150	1100	0.0105	2.33	1.80	12.5	36.9	*hooked*	0.75
	G3B1	100	200	170	1100	0.0092	2.41	1.94	12.5	36.08	*hooked*	0.5
	G3B2	100	200	170	1100	0.0092	1.29	0.82	12.5	36.08	*hooked*	0.5
	G3B3	100	275	245	1100	0.0064	0.90	0.57	12.5	36.08	*hooked*	0.5
Furlan & de Hanai 1997	*P3B*	100	100	85.25	900	0.0166	3.52	3.40	10	54.8	*crimped*	1
	P4B	100	100	85.25	900	0.0166	3.52	3.40	10	50	*crimped*	2
	P5A	100	100	85.25	900	0.0166	3.52	3.40	10	49.3	*crimped*	1
	P5B	100	100	85.25	900	0.0166	3.52	3.40	10	49.3	*crimped*	1
	P6B	100	100	85.25	900	0.0166	3.52	3.40	10	53.7	*crimped*	2
	P7A	100	100	85.25	900	0.0166	3.52	3.40	10	53.5	*crimped*	0.5
	P7B	100	100	85.25	900	0.0166	3.52	3.40	10	53.5	*crimped*	0.5
Dancygier & Savir 2011	*H3-S0-1_35*	200	325	273	2000	0.0348	2.75	2.71	22	110.9	*hooked*	0.75
	H3-S0-1_60	200	325	273	2000	0.0348	2.75	2.71	22	109.2	*hooked*	0.75
Krassowska & Kosior-Kazberuk 2018	*A-IV-WS1.0*	80	180	165	987	0.0171	2.99	2.83	4	41.23	*hooked*	1
	A-IV-WS1.5	80	180	165	987	0.0171	2.99	2.83	4	39.87	*hooked*	1.5
Yoo & Yang 2018	*S-F0.75*	300	500	420	3700	0.0322	3.21	3.19	20	62.3	*hooked*	0.75
	M-F0.75	450	750	648	5220	0.0327	3.26	3.24	20	62.3	*hooked*	0.75
	L-F0.75	600	1000	887	6780	0.0343	3.26	3.25	20	62.3	*hooked*	0.75
Gali & Subramaniam 2017	*SFRC _0.5_1*	125	250	222	1200	0.0145	1.80	1.76	10	30	*hooked*	0.5
	SFRC _0.5_2	125	250	222	1200	0.0145	1.80	1.76	10	30	*hooked*	0.5
Zamanzadeh et al. 2015	*S_W70*	70	300	270	1040	0.0332	2.56	2.52	10	50	*recycled*	0.769
	S_W110	110	300	270	1040	0.0212	2.56	2.52	10	50	*recycled*	0.769
	S_W150	150	300	270	1040	0.0155	2.56	2.52	10	50	*recycled*	0.769
Shoaib, Lubell & Bindiganavile 2014	*N31*	310	308	258	1548	0.0250	3.00	2.42	10	23	*hooked*	1
	N32	310	308	240	1440	0.0403	3.00	2.38	10	41	*hooked*	1

	H31	310	308	258	1548	0.0250	3.00	2.42	10	41	*hooked*	1
	H32	310	308	240	1440	0.0403	3.00	2.38	10	80	*hooked*	1
	N61	300	600	531	3186	0.0188	3.00	2.72	10	23	*hooked*	1
	N62	300	600	523	3138	0.0255	3.00	2.71	10	23	*hooked*	1
Shoaib, 2012	*H62*	300	600	523	3138	0.0255	3.00	2.71	10	41	*hooked*	1
	N10-1	300	1000	923	5538	0.0144	3.00	2.84	10	41	*hooked*	1
	N10-2	300	1000	920	5520	0.0203	3.00	2.84	10	41	*hooked*	1
	H10-1	300	1000	923	5538	0.0144	3.00	2.84	10	80	*hooked*	1
	H10-2	300	1000	920	5520	0.0203	3.00	2.84	10	80	*hooked*	1
Bae, Choi & Choi 2014	*U-0-f-3.5*	200	350	300	2300	0.0360	3.50	3.17	2	215	*hooked*	2
	U-0-f-2.0	200	350	300	1200	0.0360	2.00	1.67	2	199	*hooked*	2
	B2	120	300	266	1100	0.0126	1.13	1.09	20	31.9	*corrugated*	0.2
Abdul-Zaher et al. 2016	*B3*	120	300	266	1100	0.0126	1.13	1.09	20	31.9	*corrugated*	0.4
	B4	120	300	266	1100	0.0126	1.13	1.09	20	31.9	*corrugated*	0.6

Table S2: Properties of Steel Fiber

Reference	l_f/d_f	f_{tenf}	V_{utot}	v_{utot}	d_f	daggmax	F	**Failure Mode**
	**(-)**	**(MPa)**	**(kN)**	**(MPa)**	(mm)	mm		
Singh & Jain 2014	65	1100	114	3.022	0.55	12.5	0.488	DT + ST + SC
	65	1100	80	2.122	0.55	12.5	0.488	DT + ST + SC
	65	1100	110	2.922	0.55	12.5	0.650	DT + ST + SC
	65	1100	124	3.302	0.55	12.5	0.650	DT + ST + SC
	65	1100	112	2.972	0.55	12.5	0.975	DT + ST + SC
	65	1100	132	3.502	0.55	12.5	0.975	DT + ST + SC
	80	1050	66	1.742	0.75	12.5	0.400	DT + ST + SC
	80	1050	78	2.072	0.75	12.5	0.400	DT + ST + SC
	80	1050	92	2.442	0.75	12.5	0.600	DT + ST + SC
	80	1050	102	2.722	0.75	12.5	0.600	DT + ST + SC
	80	1050	117	3.102	0.75	12.5	0.800	DT + ST + SC
	80	1050	105	2.802	0.75	12.5	0.800	DT + ST + SC
	65	1100	114	3.022	0.55	12.5	0.488	DT + ST
	65	1100	127	3.362	0.55	12.5	0.488	DT + ST
	65	1100	145	3.852	0.55	12.5	0.650	DT + ST
	65	1100	166	4.422	0.55	12.5	0.650	DT + ST + SC
	65	1100	196	5.212	0.55	12.5	0.975	DT + ST
	65	1100	161	4.272	0.55	12.5	0.975	DT + ST + SC
	80	1050	128	3.412	0.75	12.5	0.400	DT + ST + SC
	80	1050	153	4.062	0.75	12.5	0.400	DT + ST + SC
	80	1050	147	3.912	0.75	12.5	0.600	DT + ST + SC
	80	1050	179	4.752	0.75	12.5	0.600	DT + ST
	80	1050	129	3.422	0.75	12.5	0.800	DT + ST + SC
	80	1050	158	4.192	0.75	12.5	0.800	DT + ST
	50	1025	80	2.112	0.6	12.5	0.375	DT + ST + SC
	50	1025	79	2.092	0.6	12.5	0.375	DT + ST + SC
	85	1050	99	2.642	0.7	12.5	0.638	DT + ST + SC
	85	1050	82	2.182	0.7	12.5	0.638	DT + ST + SC
	50	1025	100	2.662	0.6	12.5	0.375	DT + ST + SC
	50	1025	101	2.682	0.6	12.5	0.375	DT + ST
	85	1050	111	2.952	0.7	12.5	0.638	DT + ST
	85	1050	105	2.782	0.7	12.5	0.638	DT + ST
Sahoo & Sharma 2014	80	1100	144	3.679	0.75	20.0	0.400	S-FL
	80	1100	109	2.796	0.75	20.0	0.600	S
	80	1100	94	2.407	0.75	20.0	0.800	S
	80	1100	115	2.927	0.75	20.0	1.000	S
Shoaib, Lubell and Bindiganavile 2015	55	1100	204	2.549	0.5	10.0	0.550	S-FL
	55	1100	299	3.737	0.5	10.0	0.550	S-FL
	55	1100	312	1.888	0.5	10.0	0.550	S-FL
Manju et al 2017	80	1100	119	4.841	0.45	12.0	0.400	S
	80	1100	156	6.351	0.45	12.0	0.800	S
	80	1100	187	7.641	0.45	12.0	1.200	S
	80	1100	63	2.591	0.45	12.0	0.400	S

	80	1100	80	3.271	0.45	12.0	0.800	S
	80	1100	136	5.541	0.45	12.0	1.200	S
Arslan et al. 2017	55	1100	65	2.181	0.55	22.0	0.545	S
	55	1100	44	1.481	0.55	22.0	0.545	S
	55	1100	50	1.681	0.55	22.0	1.090	S
	55	1100	39	1.314	0.55	12.0	1.635	S
	55	1100	33	1.103	0.55	22.0	0.545	S
	55	1100	43	1.437	0.55	22.0	1.090	S
	55	1100	59	1.970	0.55	12.0	1.635	S
	55	1100	43	1.443	0.55	22.0	0.545	S
	55	1100	36	1.193	0.55	22.0	1.090	S-FL
Parra-Montesinos et al. 2006	80	1100	174	3.011	0.508	10.0	0.800	NA
	60	1100	151	2.601	0.508	10.0	0.900	NA
	60	1100	191	3.301	0.508	10.0	0.900	NA
	60	1100	192	3.321	0.508	10.0	0.900	NA
	80	1100	220	3.801	0.508	10.0	0.800	NA
	60	1100	198	3.411	0.508	10.0	0.900	NA
	60	1100	149	2.572	0.508	10.0	0.600	NA
	60	1100	203	3.502	0.508	10.0	0.600	NA
	60	1100	178	3.072	0.508	10.0	0.600	NA
	60	1100	181	3.132	0.508	10.0	0.600	NA
Rosenbusch & Teutsch 2003	67	1100	280	5.380	0.889	10.0	0.168	NA
	67	1100	300	5.760	0.889	10.0	0.509	NA
	67	1100	108	2.070	0.889	10.0	0.168	NA
	67	1100	144	2.770	0.889	10.0	0.509	NA
	67	1100	82	1.570	0.889	10.0	0.168	NA
	67	1100	107	2.060	0.889	10.0	0.509	NA
	67	1100	244	2.650	0.889	10.0	0.335	NA
	67	1100	252	2.740	0.889	10.0	0.335	NA
	67	1100	259	2.810	0.889	10.0	0.335	NA
	67	1100	263	2.860	0.889	10.0	0.335	NA
	67	1100	110	2.110	0.889	10.0	0.168	NA
	67	1100	120	2.310	0.889	10.0	0.342	NA
	67	1100	155	2.980	0.889	10.0	0.509	NA
	67	1100	111	2.130	0.889	10.0	0.335	NA
	67	1100	132	2.530	0.889	10.0	0.335	NA
	67	1100	153	1.420	0.889	10.0	0.168	NA
	67	1100	230	2.050	0.889	10.0	0.335	NA
	67	1100	82	1.580	0.889	10.0	0.168	NA
	67	1100	117	2.250	0.889	10.0	0.509	NA
Sahoo et al. 2016	80	1100	149	4.563	0.75	10.0	0.600	DT
	80	1100	99	3.034	0.75	10.0	0.600	SC
	80	1100	85	2.606	0.75	10.0	0.600	SC
	65	2300	286	1.532	0.9	10.0	0.209	S

Amin & Foster 2016 Tahenni et al. 2016	65	2300	356	1.907	0.9	10	0.447	S
	65	1100	42	3.124	0.54	15.0	0.325	S
	65	1100	44	3.295	0.54	15.0	0.325	S
	65	1100	43	3.199	0.54	15.0	0.325	S
	65	1100	45	3.342	0.54	15.0	0.650	S-FL
	65	1100	48	3.540	0.54	15.0	0.650	S-FL
	65	1100	43	3.187	0.54	15.0	0.650	S-FL
	80	1100	50	3.668	0.75	15.0	0.800	S-FL
	80	1100	52	3.885	0.75	15.0	0.800	S-FL
	80	1100	45	3.346	0.75	15.0	0.800	S-FL
Narayanan & Darwish 1987	100	2000	33	2.973	0.3	9.6	0.188	S
	100	2000	30	2.685	0.3	9.6	0.188	S
	100	2000	31	2.787	0.3	9.6	0.188	S
	100	2000	30	2.723	0.3	9.6	0.188	S
	100	2000	23	2.085	0.3	9.6	0.188	S
	100	2000	22	1.957	0.3	9.6	0.188	S
	133	2000	36	3.247	0.3	9.6	0.499	S
	133	2000	22	1.987	0.3	9.6	0.499	S
	100	2000	33	2.987	0.3	9.6	0.750	S
	133	2000	51	4.633	0.3	9.6	0.499	S
	133	2000	41	3.705	0.3	9.6	0.499	S
	133	2000	29	2.629	0.3	9.6	0.499	S
	133	2000	62	5.583	0.3	9.6	0.998	S
	133	2000	49	4.435	0.3	9.6	0.998	S
	133	2000	33	2.989	0.3	9.6	0.998	S
	133	2000	32	2.977	0.3	9.6	0.499	S
	133	2000	38	3.567	0.3	9.6	0.499	S
	133	2000	25	2.257	0.3	9.6	0.499	S
	133	2000	25	2.347	0.3	9.6	0.499	S
	133	2000	48	4.387	0.3	9.6	0.998	S
	133	2000	54	5.017	0.3	9.6	0.998	S
	100	2000	52	4.867	0.3	9.6	1.125	S
	100	2000	53	4.947	0.3	9.6	1.500	S
	100	2000	49	4.477	0.3	9.6	1.125	S
	100	2000	59	5.473	0.3	9.6	0.375	S
	100	2000	73	6.783	0.3	9.6	0.750	S
	100	2000	77	7.163	0.3	9.6	1.125	S
	100	2000	68	6.313	0.3	9.6	1.500	S
	60	1115	104	3.178	0.5	10.0	1.200	S
	60	1115	116	3.536	0.5	10.0	0.600	S
	60	1115	117	3.549	0.5	10.0	1.200	S
Kwak et al. 2002	63	1079	135	5.108	0.8	19.0	0.313	S-FL
	63	1079	145	5.458	0.8	19.0	0.469	S-FL
	63	1079	108	4.058	0.8	19.0	0.313	S

	63	1079	68	2.575	0.8	19.0	0.313	S
Lim & Oh 1999	60	1303	59	4.513	0.7	10.0	0.300	S
	60	1303	75	5.753	0.7	10.0	0.600	S
	55	1100	172	2.977	0.54	10.0	0.413	SC + DT + Y
	55	1100	162	2.790	0.54	10.0	0.413	SC + DT + Y
	55	1100	171	2.950	0.54	10.0	0.550	SC + DT + Y
	55	1100	174	3.003	0.54	10.0	0.550	SC + DT + Y
	55	1100	150	2.596	0.54	10.0	0.825	ST + DT + B
	55	1100	198	3.417	0.54	10.0	0.825	SC + ST
	55	1100	193	3.331	0.54	10.0	0.825	ST + DT
Dinh et al. 2010	55	1100	191	3.304	0.54	10.0	0.825	ST + DT
	80	1100	174	3.003	0.75	10.0	0.800	DT
	80	1100	220	3.805	0.75	10.0	0.800	ST + DT
	80	2300	194	3.357	0.375	10.0	0.600	ST + DT + Y
	80	2300	191	3.304	0.375	10.0	0.600	ST + DT + Y
	55	1100	369	2.952	0.54	10.0	0.413	ST + DT
	55	1100	341	2.725	0.54	10.0	0.413	DT
	80	1100	355	2.837	0.75	10.0	0.600	SC + ST
	80	1100	348	2.779	0.75	10.0	0.600	DT
	55	1100	351	2.808	0.54	10.0	0.413	SC + ST + Y
	80	1100	271	2.169	0.75	10.0	0.600	ST + DT + B
	80	1100	228	1.827	0.75	10.0	0.600	ST + DT + B
	55	1100	438	3.505	0.54	10.0	0.825	SC + ST + Y
	80	1100	424	3.393	0.75	10.0	1.200	ST + DT + Y
Lima Araujo et al. 2014	65	1150	262	5.130	0.54	12.5	0.650	NA
	65	1150	292	5.728	0.54	12.5	1.300	NA
Casanova et al. 1997	75	1200	360	3.265	0.8	12.5	0.938	NA
	60	1200	360	3.265	0.5	12.5	0.750	NA
	60	1200	158	5.610	0.5	10.0	0.750	DT
Aoude et al. 2012	55	1100	48	1.594	0.55	10.0	0.275	S
	55	1100	57	1.891	0.55	10.0	0.550	S
	55	1100	161	1.230	0.55	10.0	0.275	S
	55	1100	205	1.563	0.55		0.550	S
Minelli & Plizzari 2013	50	1100	134	1.543	0.6	20	0.190	S
	50	1100	120	1.376	1	20	0.190	S
	78	1333	142	1.629	0.73	20	0.333	S
	50	1100	141	1.623	0.6	20	0.190	S
	48	1250	191	2.198	0.62	20	0.307	S-FL
	50	1100	197	2.163	1	15	0.125	S-FL
	50	1100	157	1.724	1	15	0.125	S
	50	1100	258	1.420	1	20	0.125	S
	50	1100	339	1.865	1	20	0.125	S
Kang et al. 2011	63	1100	82	3.126	0.8	19	0.313	S-FL
	63	1100	36	1.371	0.8	19	0.313	S
	63	1100	78	2.960	0.8	19	0.313	S-FL

Casanova & Rossi 1999	60	1200	139	4.934	0.5	10	0.750	S
	60	1200	139	4.934	0.5	10	0.750	S
Lim et al. 1987	60	1130	59	1.757	0.5	10	0.300	S
	60	1130	148	4.414	0.5	10	0.600	S
	60	1130	84	2.489	0.5	10	0.600	S
	60	1130	68	2.037	0.5	10	0.600	S-FL
	60	1130	136	4.042	0.5	10	0.300	S
	60	1130	65	1.926	0.5	10	0.300	S
	60	1130	50	1.501	0.5	10	0.300	S
Mansur et al. 1986	60	1260	76	2.556	0.5	20	0.300	SC
	60	1260	53	1.800	0.5	20	0.300	SC
	60	1260	46	1.550	0.5	20	0.300	DT
	60	1260	86	2.895	0.5	20	0.450	SC
	60	1260	61	2.053	0.5	20	0.450	SC
	60	1260	66	2.223	0.5	20	0.450	SC
	60	1260	46	1.546	0.5	20	0.450	SC
	60	1260	61	2.053	0.5	20	0.450	SC
	60	1260	87	2.933	0.5	20	0.450	SC
Zarrinpour & Chao 2017	67	1096	121	3.135	0.76	10	0.503	S
	67	1096	482	3.112	0.76	10	0.503	S
	67	1096	163	2.730	0.76	10	0.503	S
	67	1096	196	3.281	0.76	10	0.503	S
	67	1096	273	2.484	0.76	10	0.503	S
	67	1096	386	3.513	0.76	10	0.503	S
	67	1096	700	3.388	0.76	10	0.503	S
	67	1096	721	3.489	0.76	10	0.503	S
	67	1096	1081	3.171	0.76	10	0.503	S
	67	1096	1044	3.063	0.76	10	0.503	S
Noghabai 2000	40	2600	300	8.326	0.15	16	0.200	S
	48	1850	296	8.215	0.375	16	0.360	S
	86	2200	253	7.021	0.7	16	0.429	S
	86	2200	263	7.299	0.7	16	0.643	S
	48	1850	190	4.865	0.375	16	0.360	S
	50	1100	311	6.616	0.6	16	0.500	S
	40	2600	364	7.744	0.15	16	0.200	S
	48	1850	408	8.680	0.375	16	0.360	S
	40	2600	293	3.570	0.15	18	0.200	S
	40	2600	340	4.143	0.15	18	0.200	S
	48	1850	371	4.521	0.375	18	0.360	S
	48	1850	331	4.034	0.375	18	0.360	S
	86	2200	268	3.265	0.7	18	0.429	S
	86	2200	316	3.851	0.7	18	0.429	S
	86	2200	343	4.180	0.7	18	0.643	S
	86	2200	296	3.607	0.7	18	0.643	S
	40	2600	458	2.679	0.15	18	0.200	S

	48	1850	609	3.562	0.375	18	0.360	S
	86	2200	522	3.053	0.7	18	0.643	S
Randl et al. 2017	75	2000	254	4.039	0.2	0.4	0.750	S
	75	2000	321	5.105	0.2	0.4	0.750	S
	75	2000	360	5.727	0.2	0.4	0.750	S
	75	2000	269	4.277	0.2	0.4	0.375	S
	75	2000	202	3.211	0.2	0.4	0.375	S
	75	2000	311	4.946	0.2	0.4	0.375	S
Ashour et al. 1992	75	260	46	1.700	0.8	10	0.750	NA
	75	260	25	0.925	0.8	10	0.750	NA
	75	260	16	0.605	0.8	10	0.750	NA
	75	260	245	9.104	0.8	10	0.375	NA
	75	260	130	4.840	0.8	10	0.375	NA
	75	260	62	2.302	0.8	10	0.375	NA
	75	260	54	1.995	0.8	10	0.375	NA
	75	260	343	12.754	0.8	10	0.750	NA
	75	260	163	6.080	0.8	10	0.750	NA
	75	260	86	3.202	0.8	10	0.750	NA
	75	260	54	2.005	0.8	10	0.750	NA
	75	260	375	13.964	0.8	10	1.125	NA
	75	260	194	7.230	0.8	10	1.125	NA
	75	260	95	3.542	0.8	10	1.125	NA
	75	260	54	2.025	0.8	10	1.125	NA
	75	260	181	6.750	0.8	10	0.750	NA
	75	260	105	3.912	0.8	10	0.750	NA
	75	260	80	2.975	0.8	10	0.750	NA
Tan et al. 1993	60	1100	219	4.606	0.5	19	0.300	NA
	60	1100	182	3.827	0.5	19	0.450	NA
	60	1100	212	4.444	0.5	19	0.600	NA
	60	1100	155	3.266	0.5	19	0.600	NA
	60	1100	308	6.476	0.5	19	0.600	NA
Pansuk et al. 2017	65	2000	342	6.505	0.2	2	0.520	NA
	65	2000	533	10.143	0.2	2	1.040	NA
Kim et al. 2017	60	1336	118	1.335	0.5	10	0.450	NA
	60	1336	208	2.354	0.5	10	0.450	NA
Sharma, 1986	85	1100	124	2.983	0.6	10	0.816	NA
Narayanan & Darwish 1988	100	2000	351	10.159	0.3	5	0.188	NA
	100	2000	326	9.435	0.3	5	0.375	NA
	100	2000	362	10.478	0.3	5	0.563	NA
	100	2000	397	11.493	0.3	5	0.750	NA
	100	2000	394	11.406	0.3	5	0.938	NA
	100	2000	455	13.174	0.3	5	0.750	NA
	100	2000	405	11.725	0.3	5	0.750	NA
	100	2000	343	9.928	0.3	5	0.750	NA
	100	2000	345	9.986	0.3	5	0.750	NA
	100	2000	295	8.536	0.3	5	0.750	NA

	100	2000	334	9.667	0.3	5	0.750	NA
Li, Ward & Hamza 1992	29	1000	17	2.560	0.877	2.36	0.214	NA
	29	1000	51	1.967	0.877	2.36	0.214	NA
	29	1000	21	3.220	0.877	2.36	0.428	NA
	29	1000	67	2.577	0.877	2.36	0.428	NA
	29	1000	18	2.750	0.877	2.36	0.214	NA
	29	1000	62	2.387	0.877	2.36	0.214	NA
	29	1000	51	7.823	0.877	2.36	0.214	NA
	29	1000	33	5.155	0.877	2.36	0.214	NA
	29	1000	30	4.646	0.877	2.36	0.214	NA
	29	1000	26	3.996	0.877	2.36	0.214	NA
	29	1000	23	3.627	0.877	2.36	0.214	NA
	29	1000	21	3.178	0.877	2.36	0.214	NA
	29	1000	18	2.759	0.877	2.36	0.214	NA
	29	1000	13	1.990	0.877	2.36	0.214	NA
	29	1000	18	2.760	0.877	2.36	0.214	NA
	57	1000	25	3.910	0.877	2.36	0.428	NA
	60	1172	79	3.067	0.5	9	0.600	NA
	60	1172	21	3.170	0.5	9	0.600	NA
	60	1172	16	2.440	0.5	9	0.600	NA
	60	1172	37	5.645	0.5	9	0.600	NA
	100	1172	79	3.067	0.5	9	1.000	NA
	100	1172	23	3.560	0.5	9	1.000	NA
Swamy et al. 1993	100	1570	81	5.538	0.5	14	0.750	S
	100	1570	59	4.077	0.5	14	0.750	S
	100	1570	43	2.945	0.5	14	0.750	S
	100	1570	72	4.962	0.5	14	0.750	S
	100	1570	46	3.158	0.5	14	0.750	S
	100	1570	43	2.965	0.5	14	0.750	S-FL
	100	1570	68	4.681	0.5	14	0.750	S-FL
Adebar et al. 1997	60	1200	278	3.306	0.5	14	0.450	S
	60	1200	326	3.878	0.5	14	0.900	S
	60	1200	206	2.449	0.5	14	0.240	S
	60	1200	234	2.782	0.5	14	0.360	S
	60	1200	249	2.961	0.5	14	0.240	S
	60	1200	239	2.842	0.5	14	0.360	S
Cho & Kim 2003	60	1100	61	3.041	0.6	13	0.300	S
	60	1100	80	3.961	0.6	13	0.600	S-FL
	60	1100	85	4.205	0.6	13	0.900	S-FL
	60	1100	95	4.747	0.6	13	0.300	S-FL
	60	1100	103	5.135	0.6	13	0.600	S-FL
	60	1100	179	8.906	0.6	13	0.300	S
	60	1100	170	8.444	0.6	13	0.600	S
	60	1100	187	9.299	0.6	13	0.900	S
	60	1100	158	7.866	0.6	13	0.300	S
	60	1100	163	8.110	0.6	13	0.600	S-FL

	60	1100	154	7.648	0.6	13	0.300	S
	60	1100	171	8.498	0.6	13	0.600	S-FL
Greenough & Nehdi 2008	50	1100	92	1.742	1	10	0.250	S
	50	1100	107	2.021	1	10	0.375	S
	50	1100	150	2.838	1	10	0.500	S
	50	1100	117	2.207	1	10	0.125	S
	50	1100	146	2.747	1	10	0.188	S
	50	1100	148	2.797	1	10	0.250	S
	43	1100	108	2.042	0.7	10	0.107	S
	43	1100	124	2.347	0.7	10	0.161	S
	43	1100	153	2.884	0.7	10	0.214	S
Kang et al. 2012	80	1100	134	2.158	0.75	9.5	0.300	S
	80	1100	223	3.911	0.75	9.5	0.300	S
Dupont & Vandewalle 2003	65	1100	111	2.141	0.92	14	0.163	S
	65	1100	121	2.334	0.92	14	0.325	S
	65	1100	156	3.007	0.92	14	0.488	S
	65	1100	282	5.418	0.92	14	0.163	S
	65	1100	302	5.802	0.92	14	0.488	S
	65	1100	84	1.607	0.92	14	0.163	S
	65	1100	110	2.094	0.92	14	0.488	S
	65	1100	110	2.110	0.92	14	0.163	S
	65	1100	146	2.802	0.92	14	0.488	S
	65	1100	84	1.620	0.92	14	0.163	S
	65	1100	119	2.283	0.92	14	0.488	S
	45	1100	102	1.941	1.11	14	0.113	S
	45	1100	122	2.323	1.11	14	0.338	S
	45	1100	102	1.956	1.11	14	0.113	S
	45	1100	122	2.341	1.11	14	0.338	S
	65	1100	132	2.514	0.92	14	0.325	S
	65	1100	159	3.062	0.92	14	0.325	S
	80	1100	149	2.848	0.63	14	0.400	S
	80	1100	165	2.702	0.75	14	0.456	S
	80	1100	165	2.702	0.75	14	0.304	S-FL
Swamy & Bahia 1985	100	1050	81	2.205	0.5	10	0.300	DT
	100	1050	116	3.144	0.5	10	0.600	S + SC
	100	1050	117	3.171	0.5	10	0.900	S + SC
	100	1050	120	3.258	0.5	10	0.600	SC + FL
	100	1050	71	1.919	0.5	10	0.600	DT
tson et al. 1972	102	1100	28	2.200	0.25	2	0.112	S-FL
	102	1100	28	2.165	0.25	2	0.112	S-FL
	102	1100	27	2.124	0.25	2	0.112	S-FL
	46	1100	28	2.220	0.41	2	0.077	S-FL
	46	1100	28	2.220	0.41	2	0.077	S-FL
	46	1100	27	2.083	0.41	2	0.077	S-FL
	102	1100	27	2.110	0.25	2	0.112	S-FL
	102	1100	32	2.489	0.25	2	0.112	S

	102	1100	32	2.483	0.25	2	0.112	S
	102	1100	28	2.200	0.25	2	0.112	S
	102	1100	25	1.986	0.25	2	0.112	S
	102	1100	30	2.331	0.25	2	0.112	S
	102	1100	28	2.207	0.25	2	0.112	S
	102	1100	33	2.593	0.25	2	0.224	S-FL
	102	1100	33	2.607	0.25	2	0.224	S
	102	1100	31	2.455	0.25	2	0.224	S
	102	1100	33	2.607	0.25	2	0.224	S
	102	1100	29	2.234	0.25	2	0.112	S
	102	1100	27	2.124	0.25	2	0.112	S
	62	1100	30	2.372	0.41	2	0.102	S
	62	1100	31	2.379	0.41	2	0.102	S
	62	1100	33	2.607	0.41	2	0.102	S
	62	1100	26	2.041	0.41	2	0.102	S
	62	1100	27	2.138	0.41	2	0.102	S
	62	1100	26	2.027	0.41	2	0.102	S
	62	1100	25	1.924	0.41	2	0.102	S
	62	1100	23	1.765	0.41	2	0.102	S
	62	1100	32	2.476	0.41	2	0.204	S
	62	1100	34	2.655	0.41	2	0.204	S
	62	1100	30	2.372	0.41	2	0.204	S
	62	1100	33	2.558	0.41	2	0.204	S
	62	1100	37	2.883	0.41	2	0.409	S
	62	1100	35	2.696	0.41	2	0.409	S
	62	1100	33	2.607	0.41	2	0.409	S
	62	1100	42	3.289	0.41	2	0.409	S
	62	1100	40	3.103	0.41	2	0.409	S
	62	1100	57	4.407	0.41	2	0.818	S-FL
	62	1100	77	6.027	0.41	2	0.818	S
	62	1100	145	11.338	0.41	2	0.818	S
	62	1100	140	10.889	0.41	2	0.818	S
	62	1100	25	1.917	0.41	2	0.102	S
	62	1100	24	1.848	0.41	2	0.102	S
	62	1100	26	2.055	0.41	2	0.102	S
Zhao et al. 2018	35	700	114	2.921	0.92	20	0.088	S
	35	700	139	3.577	0.92	20	0.175	S
	35	700	157	4.024	0.92	20	0.263	S
	35	700	150	3.853	0.92	20	0.350	S
Jindal 1984	25	4913	21	1.670	0.282	2	0.125	S
	100	2350	30	2.335	0.25	2	0.500	S
	100	2350	30	2.335	0.25	2	0.500	S
	83	2350	40	3.165	0.15	2	0.417	S
	83	2350	29	2.283	0.15	2	0.417	S
	83	2350	25	1.950	0.15	2	0.417	S
	63	2350	33	2.604	0.4	2	0.313	S

Shin, Oh & Ghosh 1994	100	1856	120	6.850	0.4	13	0.250	S
	100	1856	130	7.410	0.4	13	0.500	S
	100	1856	56	3.205	0.4	13	0.250	S
	100	1856	72	4.115	0.4	13	0.500	S
	100	1856	49	2.803	0.4	13	0.250	S
	100	1856	61	3.463	0.4	13	0.500	S
Imam, Vandewalle & Mortelmans 1994	75	2000	531	8.852	0.8	10	0.563	S-FL
	75	2000	287	4.787	0.8	10	0.563	SC
	75	2000	212	3.527	0.8	10	0.563	DT
	75	2000	215	3.578	0.8	10	0.563	DT
Huang et al. 2005 [106]	47	700	254	6.639	0.66	20	0.233	SC
Kwak, Suh & Hsu 1991	100	1100	137	3.172	0.508	9.525	1.000	S
	100	1100	146	3.379	0.508	9.525	1.000	S
	100	1100	134	3.121	0.508	9.525	2.000	S
	100	1100	139	3.224	0.508	9.525	2.000	S
Roberts & Ho 1982	100	1100	33	3.871	0.38	10	1.500	S-FL
	100	1100	36	4.271	0.38	10	2.250	S-FL
	100	1100	51	5.996	0.38	10	1.500	S-FL
	100	1100	54	6.385	0.38	10	2.250	S-FL
	100	1100	81	9.557	0.38	10	1.500	S
	100	1100	108	12.663	0.38	10	2.250	S-FL
Hwang et al. 2013	60	1200	31	1.866	0.5	10	0.300	DT
	60	1200	52	3.165	0.5	10	0.600	DT
	60	1200	54	3.285	0.5	10	0.900	DT
	60	1200	48	2.893	0.5	10	1.200	DT
	60	1200	74	4.662	0.5	10	0.600	DT
	60	1200	73	4.596	0.5	10	0.300	DT
	60	1200	82	5.127	0.5	10	0.600	SC
Spinella et al.2012	55	1100	115	3.496	0.55	10	0.545	S
	55	1100	142	4.319	0.55	10	0.545	S
Chalioris & Sfiri, 2011	75	1100	43	1.565	0.8	9.5	0.375	S
Cohen & Aoude, 2012	55	1100	44	1.670	0.55	10	0.273	S
Aoude & Cohen 2014	80	1100	46	1.733	0.375	10	0.400	S
	55	1100	45	1.712	0.55	10	0.413	S
	55	1100	59	2.245	0.55	10	0.550	S
	55	1100	60	2.283	0.55	12	0.550	S
Qissab & Salman 2018	63	1100	73	5.241	0.8	12.5	0.313	S
	63	1100	87	6.220	0.8	12.5	0.469	S
	63	1100	41	2.912	0.8	12.5	0.313	S
	63	1100	51	3.652	0.8	12.5	0.469	S-FL
	63	1100	107	7.112	0.8	12.5	0.313	S-FL
	63	1100	126	8.401	0.8	12.5	0.469	S-FL
	63	1100	45	2.981	0.8	12.5	0.313	S
	63	1100	47	3.125	0.8	12.5	0.469	S
	63	1100	42	2.489	0.8	12.5	0.313	S
	63	1100	22	1.272	0.8	12.5	0.313	S

	63	1100	51	2.100	0.8	12.5	0.313	S
Furlan & de Hanai 1997	127	1100	20	2.359	0.2	10	0.953	ST
	127	1100	22	2.594	0.2	10	1.905	S-FL
	191	1100	22	2.535	0.2	10	1.429	ST
	191	1100	19	2.183	0.2	10	1.429	ST
	191	1100	20	2.359	0.2	10	2.858	ST
	191	1100	23	2.652	0.2	10	0.714	ST
	191	1100	18	2.066	0.2	10	0.714	ST
Dancygier & Savir 2011	64	1000	202	3.704	0.55	22	0.477	S
	67	1000	211	3.863	0.9	22	0.500	S
Krassowska & Kosior-Kazberuk 2018	50	800	33	2.493	1	4	0.500	S
	50	800	41	3.138	1	4	0.750	S
Yoo & Yang 2018	65	1400	417	3.313	0.55	20	0.488	S
	65	1400	815	2.795	0.55	20	0.488	S
	65	1400	1481	2.783	0.55	20	0.488	S
Gali & Subramaniam 2017	80	1225	79	2.864	0.75	10	0.400	S
	80	1225	86	3.116	0.75	10	0.400	S
Zamanzadeh et al. 2015	58	1100	82	4.316	0.25	10	0.335	S
	58	1100	96	3.240	0.25	10	0.335	S
	58	1100	110	2.710	0.25	10	0.335	S
Shoaib, Lubell & Bindiganavile 2014	55	1100	212	2.649	0.55	10	0.545	S
	55	1100	282	3.787	0.55	10	0.545	S
Shoaib, 2012	55	1100	279	3.486	0.55	10	0.545	S-FL
	55	1100	459	6.172	0.55	10	0.545	S-FL
	55	1100	255	1.602	0.55	10	0.545	S
	55	1100	245	1.562	0.55	10	0.545	S
	55	1100	447	2.849	0.55	10	0.545	S
	55	1100	500	1.805	0.55	10	0.545	S
	55	1100	505	1.829	0.55	10	0.545	S
	55	1100	653	2.359	0.55	10	0.545	S
	55	1100	651	2.359	0.55	10	0.545	S
Bae, Choi & Choi 2014	55	1100	374	6.225	0.55	2	1.091	S
	55	1100	587	9.776	0.55	2	1.091	S
Abdul-Zaher et al. 2016	50	834	127	3.969	1	20	0.075	S
	50	834	133	4.172	1	20	0.150	S
	50	834	146	4.578	1	20	0.225	S
